# Hygroscopic movements of cone scale of white fir *Abies concolor* are tuned by quantitative variation of the scale Bauplan

**DOI:** 10.3389/fpls.2025.1603330

**Published:** 2025-05-30

**Authors:** Łukasz Wiczołek, Wiktoria Wodniok, Dorota Borowska-Wykręt, Anna Nowak, Emmanuel Arkorful, Jan J. Łyczakowski, Dorota Kwiatkowska

**Affiliations:** ^1^ Department of Plant Biotechnology, Faculty of Biochemistry, Biophysics and Biotechnology, Jagiellonian University, Kraków, Poland; ^2^ Institute of Biology, Biotechnology and Environmental Protection, Faculty of Natural Sciences, University of Silesia, Katowice, Poland

**Keywords:** cell wall, fir cone scale, hygroscopic movements, resilience, hinge-less actuator, Abies concolor, hemicellulose, ovuliferous scale Bauplan

## Abstract

Seed cones in gymnosperms consist of scales composed of dead cells at maturity. In *Abies concolor*, seed release occurs when entire seed-scale complexes, including sterile bracts that support the ovuliferous scales, are shed, causing the cone to disintegrate. This process is driven by the hygroscopic movements of the scales, which result from the reversible and uneven deformation of dead tissues in response to changes in water content. Unlike pine seed cones, which serve as a model for scale movement studies, fir features large, lamina-like ovuliferous scales that undergo extensive movements, including significant changes in surface area and profound shape transformations. Therefore, the objective of this study was to elucidate the mechanism of scale movement in fir. Quantification of surface deformation of the scale lamina and isolated tissues during transitions between dry and wet states revealed significantly higher deformation of abaxial than adaxial scale surface. Analysis of scale anatomy and chemical composition of cell walls identified three plate-shaped building blocks of the lamina: a relatively loose adaxial plate; a plate including vascular bundles built of thick-walled xylem fibers, with walls rich in xylosyl residues; an abaxial plate rich in mannosyl residues and comprising scattered sclerenchyma fibers and compact epidermis. Mechanical damaging of lamina surface and dissection of individual plates showed that lamina actuation is resilient and lamina movements are driven by interplay between the three plates. The relative plate contribution to the lamina volume tunes the extent of hygroscopic movements. In particular, different contribution of the adaxial plate to the scale thickness and related asymmetry of position of vascular bundle plate explain the profound discrepancy in the degree of scale bending despite the similarities in tissue structure, chemical composition and surface strains of individual scales. We postulate that the hygroscopic movements are tuned by simple quantitative modifications of the lamina Bauplan.

## 1 Introduction

Seed (female) cone formed by scales arranged around an elongated cone axis is typical for conifers from pine (*Pinaceae*) family. The cone scales are in fact seed-scale complexes, which comprise sterile bracts supporting seed-bearing (ovuliferous) scales. In some taxa referred to as “flexers”, the cone scales remain attached to the axis, and seeds are released in advantageous conditions by reversible movement of scales away from the axis (Losada et al., 2019). A well-known example of flexers are members of the pine genus *Pinus* L., in which the bract and ovuliferous scale are nearly completely fused. Such seed-scale complexes are tightly attached to the cone axis and bend either away from or towards the axis, depending on the external conditions. In other members of pine family referred to as “shedders”, seeds are released when the entire seed-scale complexes are shed and the cone falls apart. The shedders are represented, among others, by firs (*Abies* Mill.), in which the complexes comprise the bract and ovuliferous scales that are only partly fused, at the base, and are shed by precise abscission (localized scale dehiscence) at the connection with the cone axis (Losada et al., 2019).

The seed release in flexers and shedders is related to hygroscopic movements of the cone scales. Such movements of plant organs are due to reversible and uneven deformation of dead tissues caused by changes in water content (Elbaum and Abraham, 2014). The deformation is driven by swelling or shrinking of a cell wall matrix, which fills the space between reinforcing cellulose fibrils. Differences in such swelling or shrinking between tissues of the organ actuator lead to development of mechanical stress, the relief of which results in organ deformation (Elbaum et al., 2008; Elbaum and Abraham, 2014). The actuator usually comprises active tissue and resistance tissue that respond differently to changes in water content, the deformation of the active tissue being much stronger than that of the resistance tissue. The common configuration of the two tissues is a bilayer structure resembling a bimetallic strip (Reyssat and Mahadevan, 2009). Other actuators show a graded structure, in which active and resistance parts are not distinct. Such graded actuators comprise gradually changing tissues, from highly resisting to highly active (Borowska-Wykręt et al., 2017). Actuators are twisting, coiling or bending depending on the differences in deformation anisotropy of the two building tissue layers (Armon et al., 2011; Forterre and Dumais, 2011). Bending, which in terms of geometry is the simplest change of the actuator shape, takes place if reinforcement of the two layers is parallel or transverse to the actuator axis.

The mechanism of hygroscopic movements of pine cone scales has been extensively studied (e.g. Dawson et al., 1997; Song et al., 2015; Quan et al., 2021; Eger et al., 2022; Zhang et al., 2022). During transition from wet to dry state, the pine cone scale bends mainly in the basal region, moving the entire seed-scale complex away from the cone axis (Eger et al., 2022). The basal bending region is a bilayer actuator with the resistance layer of fibers characterized by cellulose microfibrils parallel to the scale axis, and the active layer of sclereids, the cell walls of which have cellulose microfibrils that are predominantly perpendicular to the scale axis (Dawson et al., 1997; Eger et al., 2022). Tissue at the interface between these two layers exhibits the graded structure (Quan et al., 2021). Recent studies focus also on the remaining portion of the pine cone scale. This portion undergoes minor and slow deformation while its structure is more complex than that of the basal actuator (Eger et al., 2022; Zhang et al., 2022). It is also involved in uptake of water necessary for the actuator movements (Song et al., 2015; Eger et al., 2022).

Unlike in the case of pine seed cone scales, the hygroscopic movements of seed cone scales of some fir species are not limited to the scale base. This is the case of firs with large lamina-like ovuliferous scales that are supported by small and narrow sterile bracts. Such seed-scale complex is typical of *A. alba* Mill., the fir species native to mountains throughout Europe, and often cultivated *A. concolor* (Gordon & Glend.) Lindl. ex Hildebr., which is native to mountains of western United States of America. The seed-scale complex in these firs bends not only at the base, like that in pine cone, but also the large ovuliferous scale performs extensive movements. However, the specific mechanism driving the ovuliferous scale movement in fir cones has not been fully explored. Therefore, the objective of our investigations was to elucidate the mechanism of movement of the fir ovuliferous scale, using readily available scales of *A. concolor*. We started from quantification of surface deformation of the scale lamina that accompanies transitions between dry and wet states, and identification of active and resistance tissues. Next, we examined the behavior of isolated tissues and combined the results with analysis of scale anatomy and chemical composition of cell walls. These allowed us to identify three plate-shaped building blocks of the lamina and to reveal how their relative contribution to the lamina volume tunes the extent of hygroscopic movements.

## 2 Materials and methods

### 2.1 Plant material

**Figure 1:**
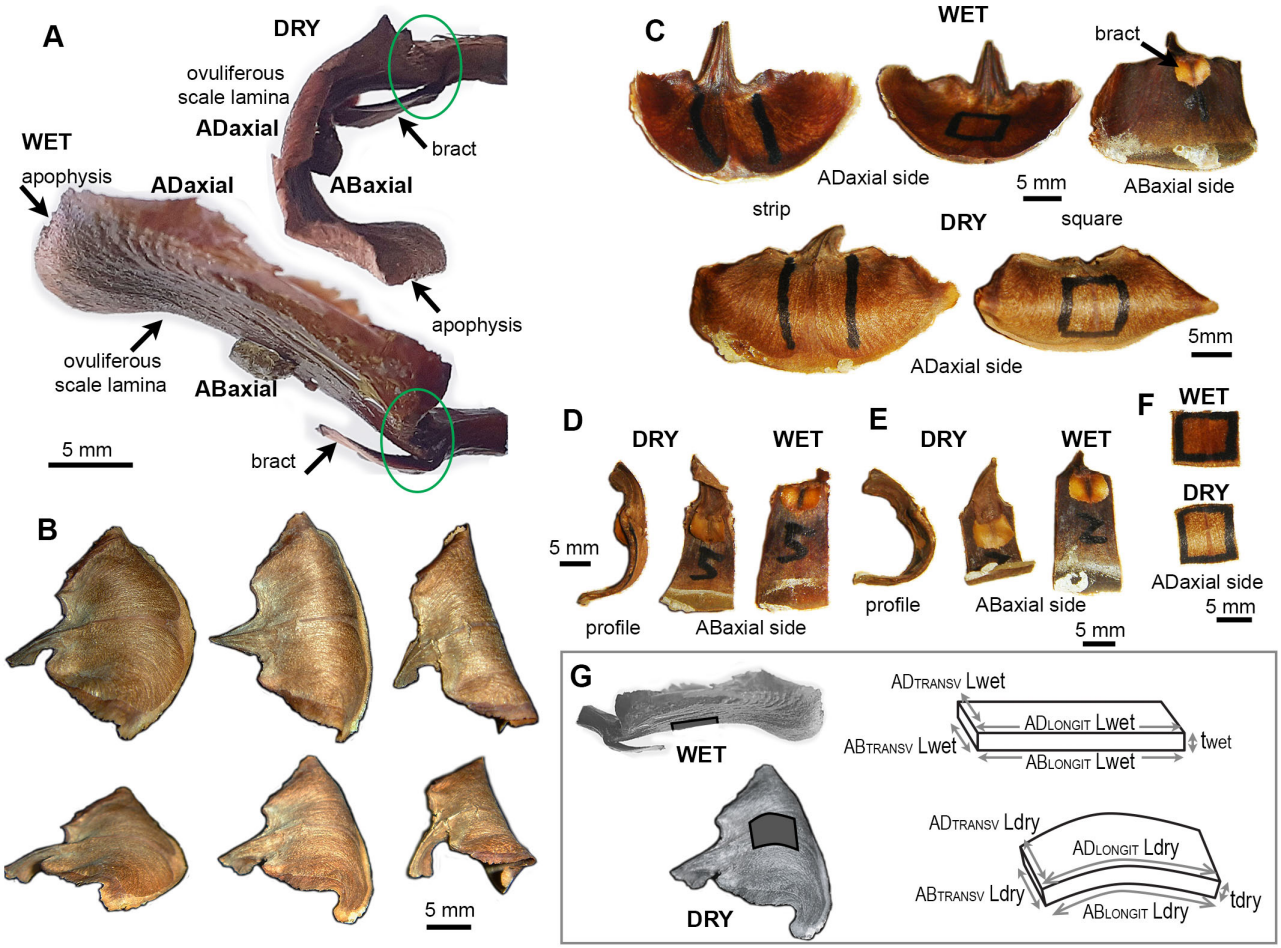
Seed-scale complex of *A. concolor* in dry and wet state: **(A)** Side views (profiles) of a seed-scale complex in dry and wet state. Two regions of the complex undergo hygroscopic movements: lamina of ovuliferous scale and the hinge at its base, outlined by green ellipse. **(B)** Three scales differing in the extent of lamina bending in dry state. The two rows show the same scales from different perspective. **(C)** Exemplary scales in wet and dry state. Two types of lamina portion used for measurements are labelled with marker on adaxial lamina surface: strip (left) and square (right). **(D, E)** Profiles and abaxial sides of lamina strips obtained from weakly **(D)** and strongly **(E)** bending scales. **(F)** Adaxial side of a lamina square in dry and wet state. **(G)** Notations used to compute strain of ovuliferous scale surface during transitions between wet and dry states. Three-dimensional schemes represent scale fragments labelled in dark gray on the images of wet and dry scales. L is the length, t – thickness, AD refers to the adaxial surface, AB – to abaxial, LONGIT represents the longitudinal direction (parallel to the scale axis), TRANSV – the transverse direction.

The seed cone scales used in this study were collected from two *Abies concolor* trees, each over 30 years old, growing on roadside in Mikołów, Silesia district, southern Poland. Mature seed-scale complexes were gathered from the ground in August-October of 2023 and August-October of 2024, having been shed naturally in 2023 and 2024, respectively. Assessment of deformation associated with transition from wet to dry state was performed on the entire ovuliferous scales using Digital Image Correlation. Strip-shaped longitudinal scale fragments (**Figure 1**), excised from wet scales and extending from the sterile bract to the ovuliferous scale margin, were used for deformation analysis via the replica method, as well as for chemical analyses, anatomical studies, and milling experiments. Additionally, square-shaped fragments of ovuliferous scales (**Figure 1**) were utilized for deformation and shape analysis, as well as milling experiments. The squares were obtained from the most curved scale portion that was identified and outlined with water-proof marker on dry scales, and cut from the scales after wetting to minimize crack formation.

### 2.2 Digital image correlation analysis of scale surface deformation

The deformation of entire ovuliferous scale surface (surface strain) was analyzed using 3D Digital Image Correlation (3D-DIC) method. Prior to measurements, scales were immersed in water for 25 min to ensure full hydration. The scale surface was then coated with a white filler spray (Filler spray, POL-EXPO sp. z o.o, Zgierz, Poland) to create a uniform background. Over this base layer, black paint speckles were applied using an aerograph (Premium color, Vallejo, Barcelona, Spain). The scale base was then stabilized with forceps in free space such that the movements of ovuliferous scale were not obstructed. Image acquisition was performed using two Marlin F-146B cameras (pixel size 4.65 μm), each equipped with 50 mm lenses (VT- LEM, Hangzhou Contrastech Co., LTD, China). The cameras were operated by Istra4D v2.3.1 (Dantec Dynamic, Skovlunde, Denmark) software. The images were captured at 15 s intervals over a 45 min period, which covered the entire drying process of the scale. Strain analysis was conducted using Istra4D v4.10 software (Dantec Dynamic, Skovlunde, Denmark). The deformation of either adaxial or abaxial surface was measured for 30 scales.

### 2.3 Assessment of deformation of adaxial and abaxial surfaces of individual scale strips using replica method

Evenly spaced 8 landmarks made with small drops of epoxy gel (High Strength 5 min Epoxy Gel, Devcon), were applied to both surfaces of dry scale strips. After complete setting of the epoxy gel, both the surfaces of the dry strip were covered with dental silicone (polyvinyl) polymer (Take 1 impression material, the hydrophilic vinyl wash material, regular set, Kerr Corp., Romulus, USA). After the polymer setting, silicone replicas of strip surface were gently removed from the strip, flattened and attached to glass slides with silicone sealant (clear, used for plumbing). The strip was then immersed in water for about 40 min, until the deformation due to wetting was complete, and the silicone replicas were acquired again from both the surfaces. The replicas were subsequently covered with a thin layer of nail polish. After drying, the nail polish film was peeled off, placed in a drop of water on a glass slide, and positioned on the black stage of a stereomicroscope (Leica S9i) for imaging with the built-in digital camera. Characteristic points of the landmarks were recognized in the images of the same surface in dry and wet state, digitized in ImageJ (https://imagej.net/ij/) and used to compute directions and values of maximal and minimal strain of scale surface accompanying transition from dry to wet state, using original script written in Python, based on the Goodall and Green (1986) method.

The strain during the transition from dry to wet state (*ϵ*) was computed as:


$$\epsilon =\frac{L_{wet}-L_{dry}}{L_{dry}}$$

where *L_wet_* and *L_dry_* are lengths in wet and dry state, respectively (**Figure 1G**).

### 2.4 Assessment of deformation of isolated tissues

Hand-cut paradermal sections of adaxial and abaxial surface, and transverse sections of the strongly bending strip portion were obtained from wet scales, placed in a drop of water on a glass slide, covered with a glass slip, and imaged with a light microscope (Nikon ECLIPSE 80i) equipped with digital camera (DS-Fi2). A second set of images was obtained from the same sections after drying for 3 h on a hot plate at 45°C. The same procedure was applied to sclerenchyma fibers isolated from abaxial side of scale strips with forceps following the removal of abaxial epidermis using a scalpel. Based on cell shapes, the same points were recognized in images of each section/fiber in dry and wet states. In paradermal sections, the points were chosen such that they marked line segments in direction parallel (longitudinal direction) or transverse (transverse direction) to scale axis recognized based on the cell wall pattern (cells or cell files are parallel to the axis). In transverse sections, the line segments were normal to the scale surface, and spanned either the entire scale thickness or the distance between the abaxial surface and the central point of vascular bundles (the center of a circle most closely resembling the shape of the bundle identified using ImageJ). Lengths of the line segments were measured using ImageJ and strains during transition from dry to wet state were computed using Matlab (Mathworks, Nattick, MA, USA).

Deformation of vascular bundles during dry to wet transition was measured using bundle segments isolated from wet scale squares by gently removing abaxial and adaxial tissues using a scalpel, and separating the bundle segments from surrounding tissue with forceps. The base of each bundle segment was attached to a glass slide using small amount of plasticine, imaged in wet, and subsequently in dry state, using a stereomicroscope (Leica M60) equipped with digital camera (IC80 HD). The length of abaxial and adaxial sides of bundles was assessed using ImageJ (segmented line tool), and strains were computed as described above.

### 2.5 Assessment of deformation of adaxial and abaxial surfaces of individual scale squares

Squares cut from weakly or strongly bending ovuliferous scales were imaged using a stereomicroscope (Leica M60) equipped with digital camera (IC80 HD). Profiles of each square side, i.e. two longitudinal profiles (of sides parallel to the scale axis) and two transverse profiles (of sides perpendicular to the axis), were imaged in both wet and dry state. The lengths of abaxial and adaxial sides of each profile was assessed using ImageJ (segmented line tool), and strains were computed for both sides as described above.

### 2.6 Dissection of plates, mechanical damage of ovuliferous scale surface, and shape documentation of dissected plates and damaged scale squares or strips

For assessment of changes of plate shapes upon dissection, first profile images of the intact scale square were acquired in dry and wet states, and then the three plates, i.e. the abaxial plate (all the tissue on the abaxial side of vascular bundles), the adaxial plate (all the tissue on the adaxial side), and the remaining plate including the bundles, were gently dissected from the square under a stereomicroscope using a scalpel and forceps. The plates were dried on a hot plate at 45°C, and their profiles were imaged again using a stereomicroscope.

Rotary milling tool (Parkside 4V Cordless Rotary Tool PFBSA 4 A1) was used to damage the adaxial or abaxial surface of the scale squares and strips. The milling was performed on wet samples. In order to analyze the milling effect on the square shape, images were taken of the square profiles (transverse and longitudinal) before and after milling, and in wet and dry state. In the case of strips, impressions of longitudinal strip profiles in plasticine were acquired before and after milling. Effects of each type of mechanical damage were examined on at least three scale squares and three strips.

### 2.7 Assessment of curvature of scale strip and square profiles

Curvature of squares was assessed for images of square profiles described above. Curvature of strips was assessed for images of strip profile impressions in plasticine acquired as scans with HP Scanjet 2400. For each profile image, points on its abaxial side were digitized using ImageJ (12–22 points for each square profile; 16–26 points for strip), and their coordinates were used to approximate the profile with a curve defined by second or third rank polynomial (the rank with higher R^2^ was chosen) using least-squares method. The curvature was then computed by differentiation based on the curve equation, for each digitized point. The mean of absolute curvature values computed for all the profile points were used for comparison of square profile shapes. All computations were performed using original code written in Matlab.

### 2.8 Light and fluorescence microscopy

Hand-cut sections were obtained from wet intact scales or from pretreated scales. During the pretreatment, scales were kept in peracetic acid (99.5% acetic acid: 30% hydrogen peroxide 1:1, v/v) for 2–3 h at 60°C and washed in water prior to sectioning. Paradermal sections were obtained from the strongly bending portion of the scale, while transverse and longitudinal sections (perpendicular or parallel to the scale axis, respectively) were taken from both the strongly bending and distal regions. Sections of intact scales were used to assess isolated tissue deformation during transition between dry and wet states; sections of intact or pretreated scales were used for analysis of scale anatomy. All the sections were examined under a light microscope (Nikon ECLIPSE 80i) equipped with digital camera (DS-Fi2).

In order to analyze the shapes of individual cells, the scale squares were dissected into three plates (abaxial plate, adaxial plate, and the remaining plate including vascular bundles), and were treated with peracetic acid overnight at 60°C. The resultant macerated tissues were washed in water, and imaged in water with a light microscope (Nikon ECLIPSE 80i) equipped with Nomarski contrast and digital camera (DS-Fi2).

Autofluorescence of scale tissues was examined in hand-cut cross-sections of strongly bending portions of intact scales using an epifluorescence microscope (Olympus BX41) equipped with digital camera (DP74) and filter cube U-MNU2 (maximum excitation wavelength 380 nm, emission 470 nm).

### 2.9 Confocal microscopy

Cross-sections of scales from the strongly bending lamina region were prepared from wet intact scales using a razor blade. Prior to each observation, sections were placed in 0.1% aqueous solution of propidium iodide (PI; Sigma-Aldrich) and stained for about 30 min under vacuum. Samples were washed in distilled water and visualized using an inverted confocal laser-scanning microscope (Olympus FV1000) equipped with objectives 10x (UPLFLN NA=0.3) or 20x (UPLFLN NA=0.5), with xyz resolution of 2.485 (µm/pixel) x 2.485(µm/pixel) x 2.0 (µm/slice) and sampling speed 8.0 µs/pixel at 10x, or xyz resolution of 1.242 (µm/pixel) x 1.242(µm/pixel) x 1 (µm/slice) and sampling speed 12.5 µs/pixel at 20x. Fluorescence excitation was performed using a 488 nm laser, and emission signal was collected at the 640 nm. Z-stacks acquired under these conditions were processed to generate images using MorphoGraphX (Barbier de Reuille et al., 2015).

### 2.10 Scanning electron microscopy

Vascular bundles with surrounding tissue, isolated from the strongly bending lamina region, and paradermal sections of adaxial and abaxial scale surfaces were examined in scanning electron microscope (SEM). Prior to the examination, the vascular bundles were treated with peracetic acid for 2 h at 60°C, washed in water, and cut by oblique plane. Paradermal sections and sections of vascular bundles were fixed in 100% methanol for 30 min. A vacuum infiltration was applied in cases where the samples did not submerge. The samples were then dehydrated twice in 100% dry ethanol, each for 30 min. After the dehydration, a critical point drying procedure was applied using EM CPD300 Automated Critical Point Dryer (Leica), following the ‘Rice Root’ protocol according to manufacturer’s recommendation. Prior to imaging, the samples were mounted on SEM stubs, coated with 10 nm of gold using Safematic CCU-010 high vacuum coating unit (Safematic GmbH, Zizers, Switzerland), and stored in a desiccator. The samples were examined in field emission SEM (UHR FE-SEM Hitachi SU 8010) at accelerating voltage of 10 kV.

### 2.11 μ-CT

X-ray microtomography (μ-CT) was used to analyze the internal three-dimensional structure of intact scales. All the scans were acquired with GE Phoenix v|tome|x system (General Electric Sensing and Inspection Technologies/Phoenix X-ray, Wunstorf, Germany). X-ray tube voltage of 100 kV and current of 150 μA were applied to capture 1800 projection images with an individual exposure time of 131 ms during 20 min scan time. The images were captured with a resolution of 2024 x 2024 pixels. The reconstruction and 3D visualization were performed using myVGL 3.4.0 software (Volume Graphics GmbH, Hedeilberg, Germany).

### 2.12 Sample preparation for biochemical analyses

Biochemical analyses were performed for strips of ovuliferous scales from which the apophysis was removed (rectangular scale fragment was excised from the middle of each prewetted scale, with a width corresponding to that of a bract scale and a length extending from the base to just before the curved margin). Under a stereoscopic microscope, vascular bundles, as well as the adaxial and abaxial plates of each scale, were carefully separated using a scalpel and tweezers.

Cell wall materials were extracted separately from abaxial and adaxial plates, as well as vascular bundles, following the alcohol-insoluble residue (AIR) method described by Goubet et al. (2009). Briefly, the tissue was homogenized using a mortar and pestle, suspended in 96% ethanol, and centrifuged at 4000 × g for 15 min. The pellet was washed with 96% ethanol and subsequently incubated overnight in a chloroform: methanol (2:1, v/v) solution. Following centrifugation and an additional wash with chloroform: methanol solution for 1 h, the pellet was sequentially washed with 65%, 80%, and 96% (v/v) ethanol. The resulting AIR was air-dried at 37°C before further analysis.

### 2.13 Biochemical analyses

For monosaccharide composition analysis, a total of 1 mg of AIR was hydrolyzed in 500 µL of 2 M trifluoroacetic acid (TFA) at 120°C for 2 h. Following hydrolysis, samples were centrifuged at 16000 × g for 5 minutes, and 400 µL of the monosaccharide-containing supernatant was evaporated under a nitrogen stream. Monosaccharides were subsequently derivatized using 1-phenyl-3-methylpyrazol-5-one (PMP) (Sigma-Aldrich, M70800). For each reaction, 25 µL of 0.5 M PMP in methanol, 15 µL of 0.5 M NaOH and 10 µL of ultrapure water were added to the dried sample, followed by incubation at 70°C for 2 h, and the reaction was subsequently neutralized with 20 µL of 0.5 M HCl. Excess PMP was removed by extracting twice with 600 µL of chloroform. The remaining aqueous phase was concentrated using a vacuum concentrator, resuspended in 250 µL of ultrapure water, and filtered through a 0.45 µm PTFE membrane (Pall AcroPrep 96-well filter plate). For HPLC analysis (adapted from Yu et al., 2014; Liszka et al., 2023), 50 µL of the PMP-derivatized monosaccharides were injected onto a Phenomenex Fusion-RP column connected to an Agilent 1260 Infinity II HPLC system (Agilent Technologies, USA). Compounds were eluted isocratically using a mobile phase of 100 mM phosphate buffer (pH 6.6) and acetonitrile (82:18, v/v) at a flow rate of 1 mL/min. Monosaccharide standards, including L-fucose, L-rhamnose, L-arabinose, D-glucose, D-galactose, D-mannose, D-xylose, D-glucuronic acid, and D-galacturonic acid, were used for peak annotation. Monosaccharide content was quantified based on the area under each peak. The analysis was conducted on 5–6 biological replicates (for strongly bending scales: 5 for adaxial tissue, 5 for vascular bundles, 6 for abaxial tissue; for weakly bending: 6, 5, and 6, respectively) with each replicate analyzed in triplicate.

For crystalline cellulose content analysis, a 1 mg sample of AIR was hydrolyzed in 500 μL of 2 M trifluoroacetic acid at 120°C for 2 h. Following hydrolysis, the samples were centrifuged for 10 minutes, and the resulting pellet was washed thrice with ultrapure water and dried (Liszka et al., 2023). The dried pellet was subjected to a two-step hydrolysis using sulfuric acid to release glucose (after Fang et al., 2016), and the resultant hydrolysate was assayed for crystalline cellulose content using anthrone assay as described by Kumar and Turner (2015). A glucose standard curve was generated to determine cellulose concentration in the samples.

### 2.14 Software used for statistical analysis and image processing

Statistical analysis was performed using Statistics Toolbox of Matlab and RStudio (R version 4.4.2), artwork preparation was performed using Adobe Design Premium CS4 (Adobe Systems Inc., USA).

## 3 Results

### 3.1 Both the base and lamina of the ovuliferous scale of *Abies concolor* undergo profound hygroscopic movements

The seed-scale complex in *Abies concolor* comprises a small bifurcate sterile bract that supports a much bigger ovuliferous scale (**Figure 1A**), which has a shape of reniform lamina (**Figures 1B, C**). The distal lamina margin, i.e. apophysis, is the scale portion, which is exposed on the cone surface when the scale remains attached to the cone axis, and is often covered with resin. The hygroscopic movements of the seed-scale complex during the transition between dry and wet states take place in two regions (**Figure 1A**). Near the base of complex, there is a hinge-like region of rather strong bending (outlined by green ellipse in **Figure 1A**). This bending results in a swaying-like movement of the lamina of ovuliferous scale. Simultaneously, the transitions between the dry and wet states lead to profound changes in the three-dimensional shape of lamina (**Figures 1A, C**). In the dry state, the lamina resembles a saddle: it is strongly convex along the longitudinal scale axis, and slightly concave in the transverse direction (**Figures 1A, B**). In the wet state, the lamina shape is trough-like: it is nearly straight along the longitudinal axis and concave in the transverse direction (**Figures 1A, C** – upper row). Noteworthy, while the lamina shapes of various scales in the wet state are similar (**Figure 1C** – upper row) there is an apparent shape variation in the dry state, i.e. in the extent to which the lamina is curved along the longitudinal scale axis (compare the nearly flat and strongly curved laminas in **Figure 1B** and profiles of lamina strips in **Figures 1D, E**). We focused on the mechanism of these profound changes of the lamina shape and the variation between individual scales.

**Figure 2:**
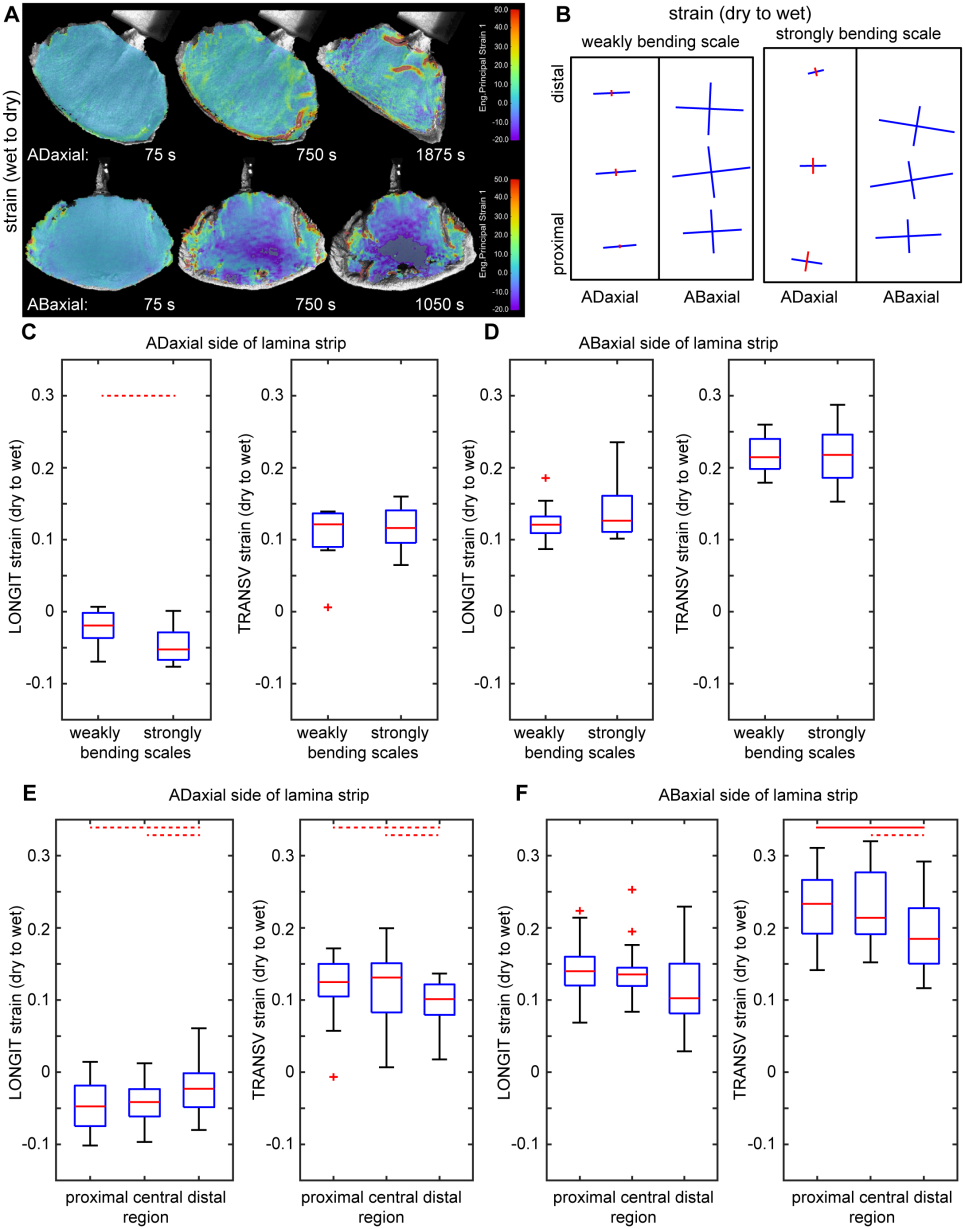
Surface deformation of ovuliferous scale lamina during transitions between dry and wet states: **(A)** Colormap representing surface deformation of lamina assessed using Digital Image Correlation. Each image shows strain in the maximal strain direction computed for a time interval preceding time-points given below each map. The dark grey color, in the central part of the map of abaxial surface for the last time-point, means that the strong shrinking in this region is out of the colormap scale. **(B)** Maps of deformation of adaxial and abaxial surfaces of lamina strip during transition from dry to wet state, assessed using the replica method. Crosses show directions of maximal and minimal deformation, the arm lengths are proportional to strain in the arm direction, blue color represents extension, red – shrinking. **(C–F)** Surface strains during transition from dry to wet state in the directions parallel (LONGIT strain) and transverse (TRANSV strain) to the scale axis, assessed using the replica method for adaxial and abaxial surfaces of lamina strips. Comparison between weakly and strongly bending scales **(C, D)** and between regions within scales **(E, F)** is presented. The boxes delimit the first and third quantiles, whiskers extend from each end of the box to the adjacent values in the data as long as the most extreme values are within 1.5 times the interquartile range from the ends of the box. Crosses are outliers; red line segments within boxes mark medians. Statistically significant differences between the variable means, assessed using Student t-test are marked by dashed (0.01< p< 0.05) or solid (p =< 0.01) lines. See **Supplementary Table S2** for t-test results.

First, we used Digital Image Correlation (DIC) to assess deformation of the entire adaxial and abaxial surfaces of the lamina during the transition from wet to dry state, and showed that the deformation differs between the two surfaces (**Figure 2A**). The strain in the maximal strain direction is low and rather uniform on the adaxial surface while on the abaxial surface the absolute strain values are higher, especially in the strip-shaped central portion of lamina located between the seed-scale complex base and apophysis (outlined in **Figures 1C–E**). We therefore used the replica method to assess the deformation during the transition from dry to wet states for such strip-shaped central portions of the lamina (**Figures 2B–F**; **Supplementary Figure S1**). Because the hygroscopic movements are driven by differential deformation of the organ tissues, we assessed the deformation of adaxial and abaxial surfaces of individual scale strips. Indeed, examination of six such scale strips confirmed the difference in deformation of the opposite surfaces (**Figures 2C, D**). During wetting, the abaxial surface expands anisotropically with stronger expansion in the transverse direction (compare the arms of crosses representing directions of maximal and minimal strain in **Figure 2B**). The anisotropy of adaxial surface deformation is much stronger: during wetting the surface shrinks in the longitudinal direction (along the scale axis; red cross arms in **Figure 2B**) and expands in the transverse direction (blue arms in **Figure 2B**). The extent of expansion in the transverse direction is higher on the abaxial surface than on the adaxial (**Figures 2C, D**; **Supplementary Tables S1, S2**). Similarly, the extent of abaxial surface expansion in the longitudinal direction is higher than the absolute value of the adaxial surface shrinking in the same direction. Thus, the active tissue is located on the abaxial side of the lamina while the adaxial side can be regarded as the resistant tissue. Surprisingly, the differences between deformation of the abaxial and adaxial surfaces are similar in strongly and weakly bending scales (**Figures 2C, D**; **Supplementary Tables S1, S2**).

The deformation assessment using replicas also reveals a strain gradient along the lamina axis: the absolute strain values in the distal lamina region (near the apophysis) are significantly lower than those in the middle and proximal regions, except for the longitudinal strain on the abaxial surface, which deviates from this pattern (**Figures 2E, F**; **Supplementary Tables S1, S2**). The lamina regions with higher strains coincide with the part that is relatively strongly curved in the longitudinal direction in the dry state (**Figures 1A, D, E**). Thus, for further analyses, besides the lamina strips, we used also lamina portions of square shape, that were obtained from this relatively strongly curved region, which was identified in the dry state (outlined in **Figures 1C, F**).

### 3.2 Dead cells of various shape and wall thickness are assembled in three main building blocks (plates) of ovuliferous scale lamina

**Figure 3:**
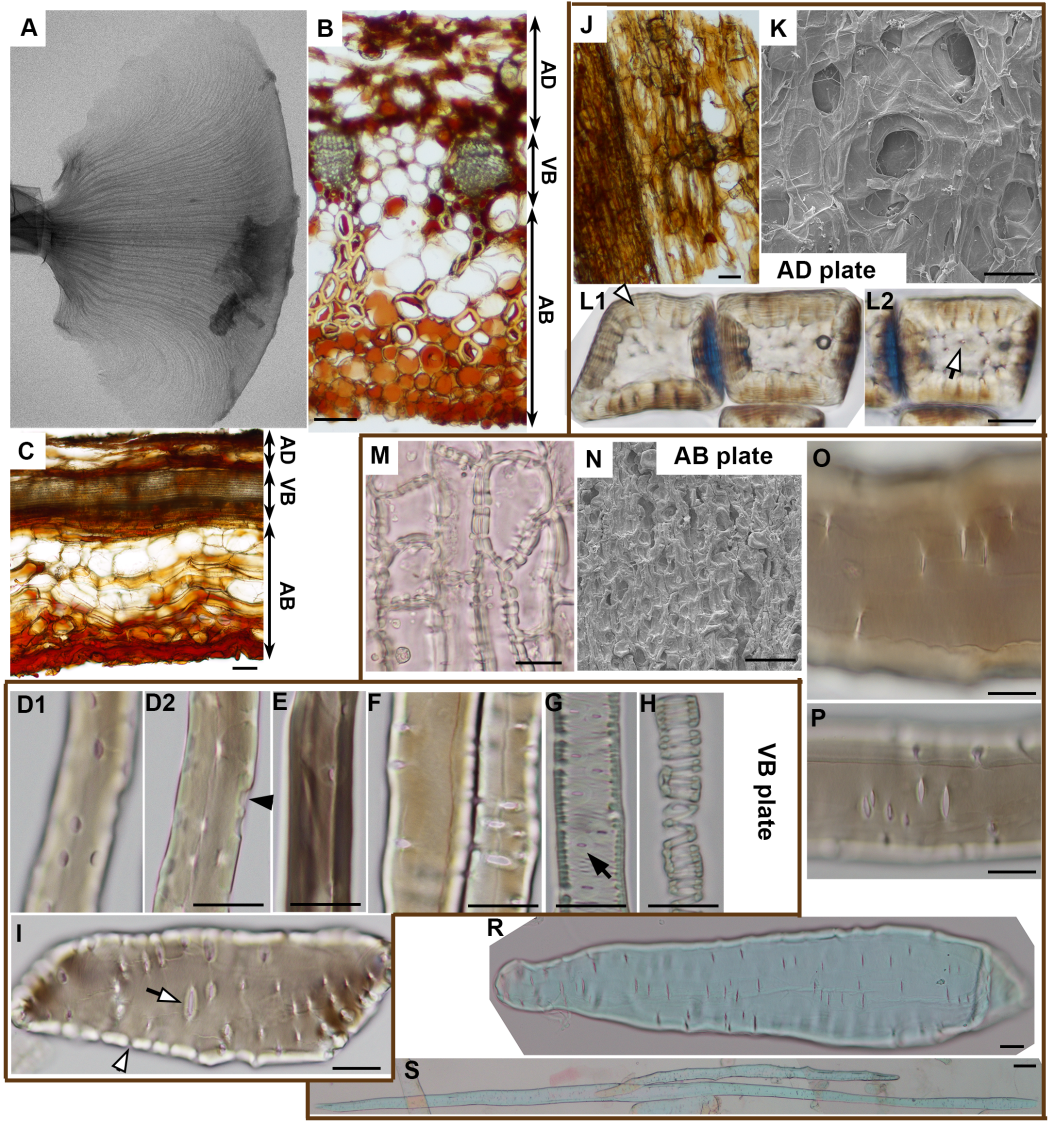
Anatomy of ovuliferous scale lamina in *A. concolor*: **(A)** Microtomography image of the entire seed-scale complex showing VB scaffold (fan-shaped pattern of darker thin strands). **(B, C).** Transverse **(B)** and longitudinal **(C)** sections from strongly deforming region. Three plates are delineated (AD – adaxial plate; VB – VB plate, AB – abaxial plate). **(D–I)** Cells building VB plate obtained by tissue maceration: fiber-tracheids and tracheids **(D–H)**, sclereid **(I)**. **(D1-2)** are optical sections of the same fiber-tracheid with bordered pits, the inner apertures of which are slightly elongated in longitudinal direction; a pit chamber typical for bordered pits is pointed by black arrowhead; **(E)** shows a fiber-tracheid with bordered pits with rounded apertures; **(F)** two tracheids with bordered pits, the apertures of which are elongated in transverse direction; **(G, H)** tracheids with helical tertiary thickenings and bordered pits with apertures elongated in direction transverse to cell and scale axis, pointed by arrow; **(I)** sclereid with simple pits (white arrowhead points to a straight pit canal) the apertures of which are elongated in direction transverse to cell and scale axis. **(J–L)** Adaxial plate: paradermal section with a patch of continuous epidermis (on left) and exposed parenchyma observed in light microscope **(J)**; adaxial surface observed in SEM **(K)**; **(L1-2)** are two optical sections of sclereids with simple pits (pointed by white arrowhead and white arrow) in thick cell walls. **(M–S)** Abaxial plate: paradermal section of continuous abaxial epidermis observed in light microscope **(M)**; abaxial surface observed in SEM **(N)**; **(O, P)** show fragments of a parenchyma cell **(O)** and a sclerenchyma fiber **(P)** with simple pits the apertures of which are elongated in direction transverse to cell and scale axis; **(R)** is an entire parenchyma cell; **(S)** an entire sclerenchyma fiber, both of them are elongated in direction of scale axis. Scale bars are 100 µm **(B, C, J, K, N, S)** or 20 µm **(D–I, L, M, O–R)**.

**Figure 4:**
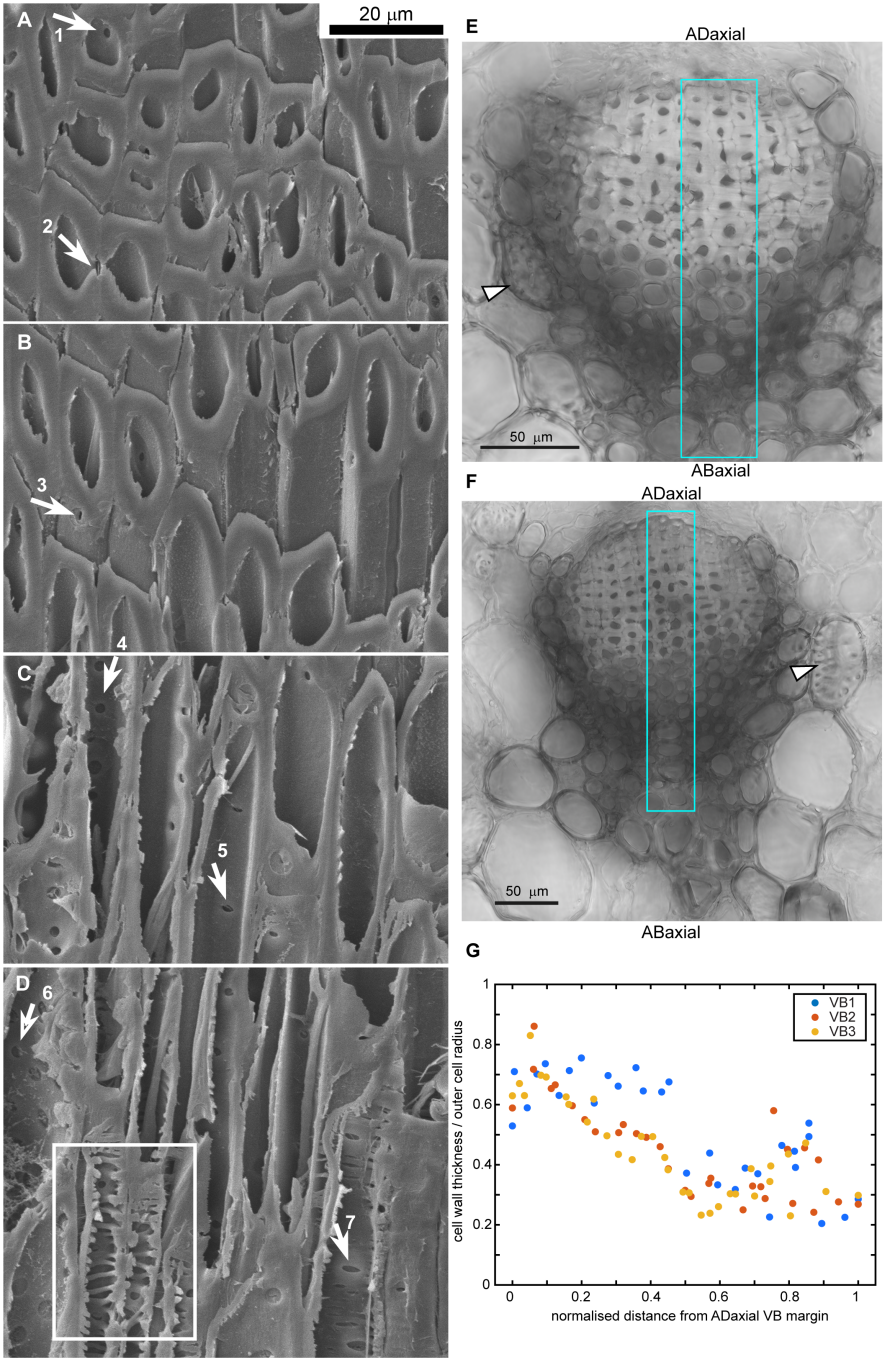
Gradient of cell wall structure and density in vascular bundles (VB) of strongly bending lamina region: **(A–D)** SEM micrographs of oblique section of VB, partially hydrolyzed prior to sectioning. The images are ordered from the adaxial **(A)** to abaxial **(D)** boundaries of the VB. Arrows point to pits in cell walls of fiber-tracheids and tracheids: 1-2,4 – bordered pits with rounded aperture, 3 – bordered pit with aperture elongated in longitudinal direction, 5,7 – bordered pits with aperture elongated in transverse direction, 6 – simple pit with rounded aperture. Exposed inner surface of wall decorated with helical tertiary thickenings is outlined in **(D)**. **(E, F)** Cross-sections of VBs prepared by compilation of 9 **(E)** or 3 **(F)** optical sections obtained from confocal microscopy (bright field images). Sclereids of transfusion tissue are pointed by arrowheads. Regions used for wall density assessment are outlined in blue. **(G)** Cell wall density, computed as the ratio of cell wall thickness divided by the outer cell radius, is plotted against the normalized distance from the adaxial VB margin for three individual VBs. Section of VB1 is shown in **(E)**, VB3 – in **(F)**.

Having identified the active and resistant sides of the ovuliferous scale lamina, we investigated the anatomy of the lamina to elucidate the differences between the adaxial and abaxial tissues (**Figures 3**, **4**). The scale is scarious, i.e. built exclusively of dead tissues (sclerenchyma, xylem, parenchyma, epidermis). X-ray microtomography revealed a fan-like scaffold formed by vascular bundles (VBs) spreading from the scale base to margins (**Figure 3A**; **Supplementary Figure S2**). Each VB comprises a compact (lacking intercellular gas spaces, see **Figures 4E, F**) bundle of thick-walled fiber-tracheids and tracheids that is surrounded by scattered sclereids with thinner walls (pointed by arrowhead in **Figures 4E, F**). These sclereids are large, only slightly elongated cells and can be regarded as elements of the transfusion tissue that typically surrounds vascular bundles of gymnosperm leaves (Kaniewski and Kucewicz, 1978; Romberger et al., 1993). Walls of both fiber-tracheids and tracheids have bordered pits typical for secondary xylem elements (**Figures 3D–G**; pointed by arrows 1-5, 7 in **Figures 4A–D**) while the pits in sclereid walls are simple (**Figure 3I**; pointed by arrow 6 in **Figure 4D**). A small group of tracheids in the abaxial VB region have thinner secondary walls with distinct bordered pits (pointed by arrow in **Figure 3G**) and helical tertiary wall thickenings (**Figures 3G, H**; outlined in white in **Figure 4D**). Phloem elements, which are formed on the adaxial side of VB during the scale development (Kaniewski and Kucewicz, 1978), cannot be identified probably because they are damaged during the process of scale drying upon maturation. The structure of the VBs changes along the adaxial-abaxial axis. First, there is an adaxial-abaxial gradient in cell wall density, which decreases towards the abaxial VBs margin (**Figures 4E–G**). Moreover, the shape of bordered pit apertures is not uniform: they are rounded (**Figure 3E**; pointed by arrow 1 in **Figure 4A**) or slightly elongated in longitudinal direction (**Figure 3D1**; pointed by arrow 3 in **Figure 4B**) on adaxial side of the VBs and elongated in transverse direction (**Figures 3F, G**; pointed by arrows 5, 7 in **Figures 4C, D**) on the abaxial VB side. Pits with rounded apertures (pointed by arrow 6 in **Figure 4D**) or with apertures elongated in transverse direction are present also in walls of sclereids (**Figure 3I**). The shape of pit aperture is usually related to the major orientation of cellulose fibrils (Cockrell, 1974; Lichtenegger et al., 2003). Thus, we conclude that the fibrils orientation in adaxial part of VBs is nearly longitudinal while it is nearly transverse in the abaxial VB part.

The VBs surrounded by putative transfusion tissue are embedded in parenchyma built of thinner-walled cells (**Figures 3B, C**). Resin canals are scattered between the VBs. The tissue on the adaxial side of the VBs is built of relatively thin-walled large parenchyma cells (**Figures 3B, C**) in which groups of thick-walled sclereids (**Figure 3L**) are embedded. Because seed wings originate from the outer cell layers of the adaxial tissue (Kaniewski and Kucewicz, 1978; Kolotelo, 1997), the adaxial lamina surface is only partly covered with epidermis while in remaining places the parenchyma and sclereids are exposed (**Figures 3J, K**). The tissue on the abaxial side of the VBs is composed of numerous very long sclerenchyma fibers (**Figures 3B, S**) with simple pits, the apertures of which are elongated in transverse direction (**Figure 3P**). The abaxial sclerenchyma fibers are scattered between large thin-walled parenchyma cells of similar pit shapes (**Figures 3O, R**), together with resin canals. The abaxial lamina surface is covered by compact layer of thick-walled abaxial epidermis (**Figures 3M, N**).

Thus, the lamina construction (Bauplan) resembles a sandwich: the VBs extend in a plate, which is located between two rather thick plates of other tissues, one on the adaxial and the other on the abaxial side (**Figures 3B, C**). Boundaries between these three plates are not distinct but the plates are distinguished by their contributing tissues and the position with respect to the VBs. Accordingly, the plate-shaped lamina portion, including the VBs, parenchyma and resin canals between the VBs, will be hereafter referred to as the ‘VB plate’. The tissue on the adaxial side of the VBs, including parenchyma with groups of sclereids, partly covered with epidermis, will be called the ‘adaxial plate’; the tissue on the abaxial side of VBs, including parenchyma with sclerenchyma fibers, covered by continuous epidermis – the ‘abaxial plate’. Noteworthy, the location of VB plate is asymmetric, i.e. the VBs are closer to the adaxial than to the abaxial lamina surface, i.e. the adaxial plate is thinner than the abaxial (**Figures 3B, C**).

### 3.3 Isolated scale tissues differ in deformation during transition between wet and dry states

**Figure 5:**
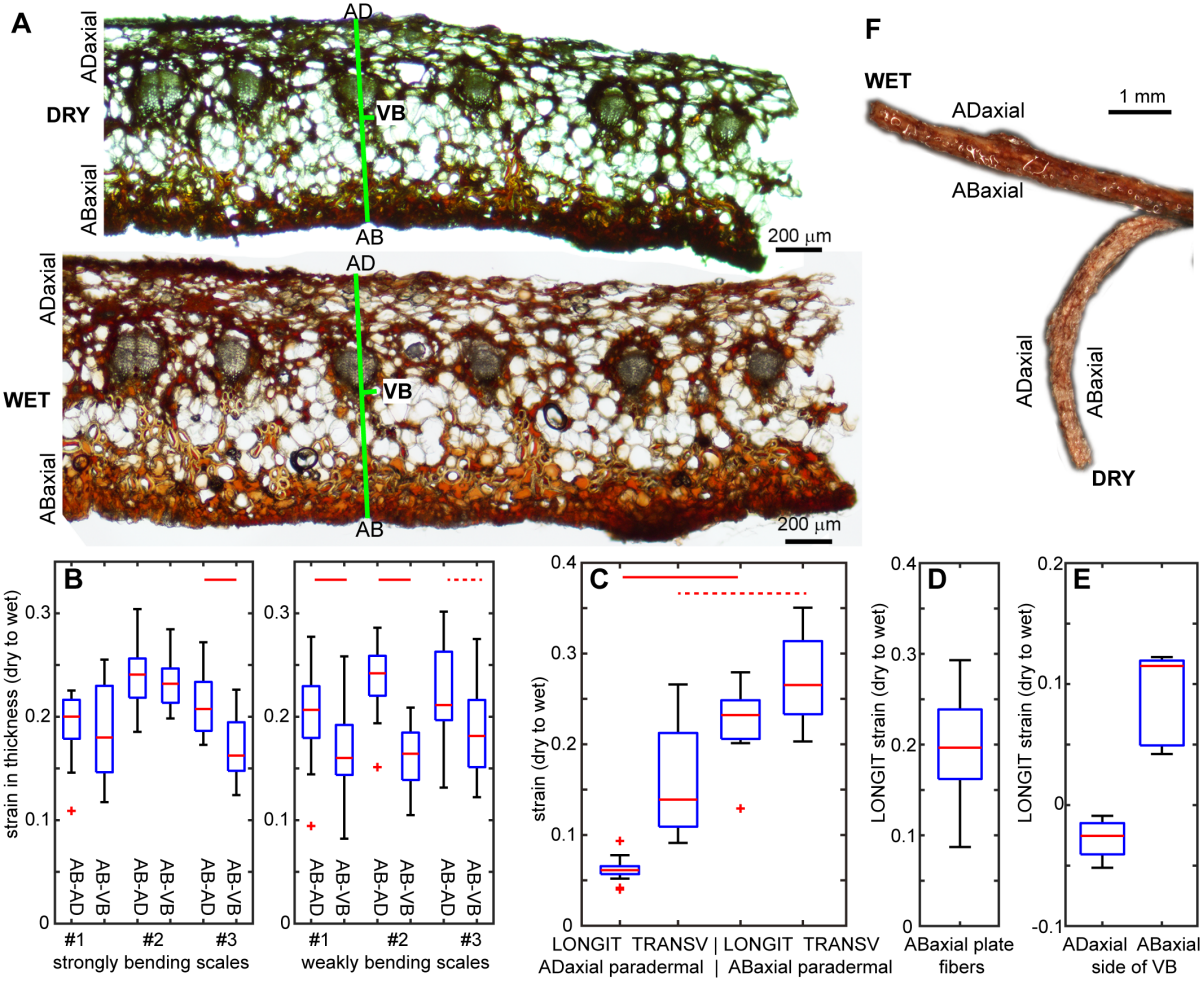
Deformation of isolated scale tissues during transition from dry to wet state: **(A)** Cross-section of strongly bending lamina region in dry and wet state. Note the apparent increase in the entire lamina thickness (labelled as AB-AD) and in the thickness of abaxial plate (AB-VB). **(B)** Strain in thickness assessed from cross-sections like that shown in **(A)**, for the entire lamina thickness and for the abaxial plate. **(C)** Longitudinal strains (LONGIT, i.e. in the direction parallel to scale axis) and transverse strains (TRANSV, i.e. in the direction transverse to the scale axis), assessed for paradermal sections of adaxial and abaxial lamina surfaces. **(D)** Longitudinal strain (extension) of sclerenchyma fibers isolated from the abaxial plate. **(E)** Longitudinal strain (positive – extension or negative – shrinking) of abaxial and abaxial sides of isolated VBs. **(F)** Isolated VB in dry and wet state. See **Figure 2** legend for further explanations. See **Supplementary Table S2** for t-test results.

We isolated scale tissues from different plates and compared their deformation during the transition from dry to wet state (**Figure 5**). First, we assessed changes in tissue thickness using hand-cut cross sections isolated from the lamina region performing the strong hygroscopic movements (strongly curved in dry state). The lamina thickness increases significantly during wetting (**Figures 5A, B**). The strain in thickness of the abaxial plate (between labels AB and VB in **Figure 5A**) is slightly lower than that of the three plates put together, i.e. entire lamina thickness (between labels AB and AD). This difference is statistically significant in all the examined weakly bending scales and in one out of three strongly bending scales (**Figure 5B**, **Supplementary Tables S1, S2**).

Next, the deformation in directions parallel to the lamina surface was assessed for hand-cut paradermal sections (sections parallel to the lamina surface) of adaxial and abaxial tissues, and for sclerenchyma fibers isolated from the abaxial plate (**Supplementary Figure S3**). There are statistically significant differences between deformation of the paradermal sections of the abaxial and adaxial tissues (**Figure 5C**; **Supplementary Tables S1, S2**), similar to differences between deformation of the abaxial and adaxial lamina surfaces *in situ*, i.e. deformation assessed for replicas of lamina strips. Both longitudinal and transverse surface strains are higher in abaxial than adaxial tissue but strain anisotropy, i.e. difference between longitudinal and transverse strains, is lower for the abaxial tissue than for the adaxial (**Figure 5C**). Adaxial tissue expands much stronger in the transverse than in the longitudinal direction, although unlike *in situ*, it is not shrinking in the longitudinal direction. In the case of abaxial tissue, there is strong expansion in both the directions but the strain is larger in transverse than in longitudinal direction, similar to strains *in situ*. Isolated abaxial fibers extend upon wetting, with strain values similar to the longitudinal strain of the abaxial tissue (**Figure 5D**). In order to assess the strain of the VBs, we analyzed segments of the entire VBs, which comprised bundles of fiber-tracheids and tracheids covered with sclereids. The segments were isolated from the lamina region that performs strong movements. During the transition from dry to wet state, the shape of the VB segments changed from curved to straight, which was accompanied by extension of the abaxial VB side and slight contraction of the adaxial side (**Figures 5E, F**).

### 3.4 Differences in tissue deformation during transitions between dry and wet states are related to cell wall composition

**Figure 6:**
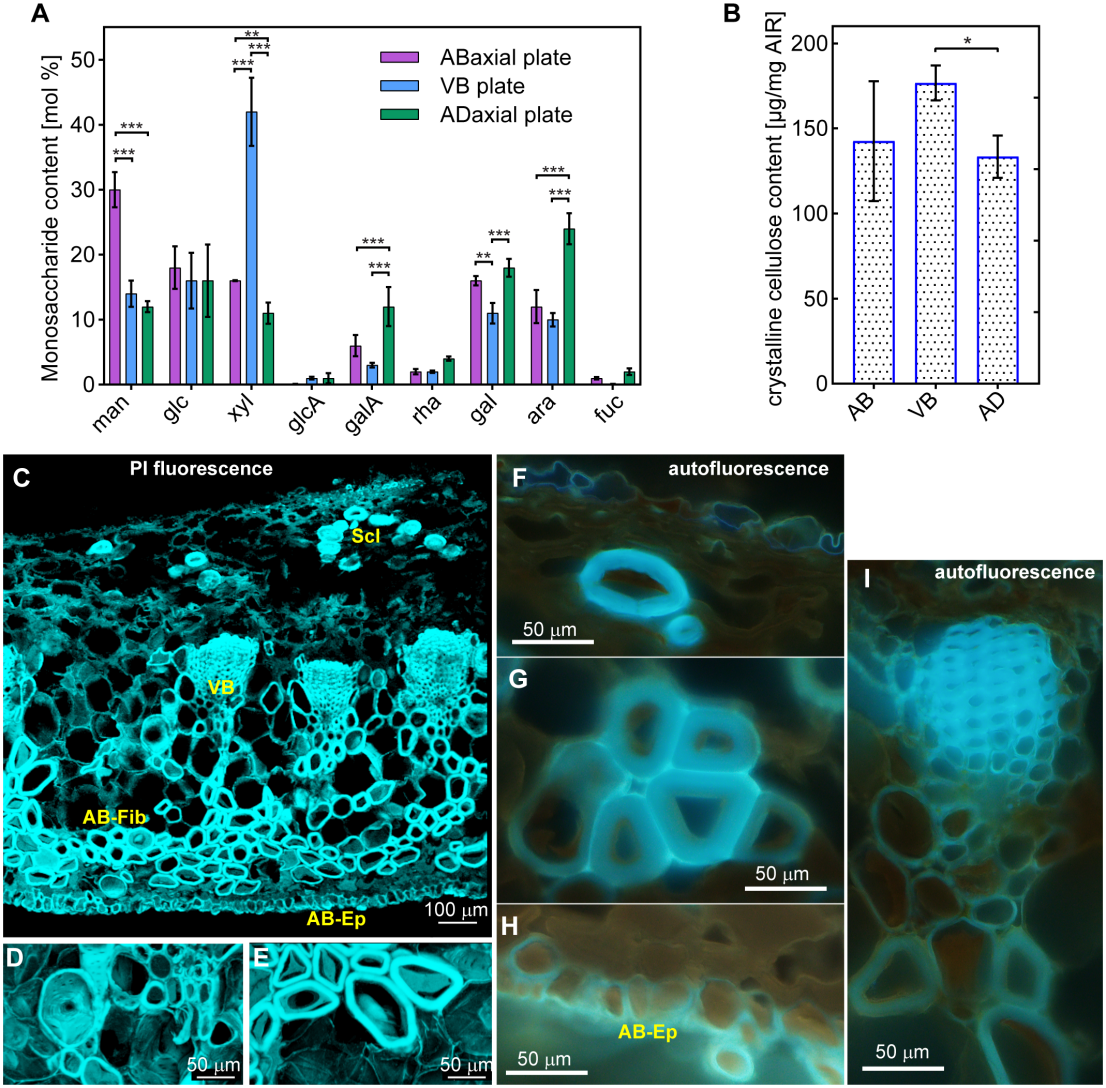
Chemical composition of cell walls of adaxial (AD), VB, and abaxial (AB) plates: **(A)** Monosaccharide content (man – mannose, glc – glucose; xyl – xylose, glcA – glucuronic acid, galA – galacturonic acid, rha – rhamnose, gal – galactose, ara – arabinose, fuc – fucose). **(B)** Crystalline cellulose content. See **Supplementary Table S3** for statistical results. *** indicates p =< 0.001, ** indicates 0.001 < p =< 0.01, * indicates 0.01 < p =< 0.05. **(C–E)** Cross sections of the strongly bending scale region stained with PI, obtained from confocal stacks using MorphoGraphX, showing entire cross-section of the scale **(C)**, transfusion tissue sclereids adjacent to the VB **(D)**, and abaxial sclerenchyma fibers **(E)**. Note that the blue color is artificial and not the real color of the emitted light. **(F–I)** UV induced autofluorescence of cell walls in cross sections of the strongly bending scale region: adaxial epidermis and sclereid **(F)**, abaxial sclerenchyma fibers **(G)**, abaxial epidermis **(H)**, and vascular bundle **(I)**. Sclereids of adaxial plate (Scl), a vascular bundle (VB), abaxial fibers (AB-Fib), and abaxial epidermis (AB-Ep) are labelled.

To evaluate the contribution of cell wall biochemistry to the scale lamina movement, we isolated alcohol-insoluble residue (AIR) cell wall materials from the VB, abaxial and adaxial lamina plates. Monosaccharide composition analysis of cell wall material revealed substantial differences between the three plates (**Figure 6A**; **Supplementary Table S3**). The thick cell walls of vascular bundle cells were predominantly composed of carbohydrates rich in xylosyl residues. In contrast, cell wall materials from the abaxial side, which is abundant in sclerenchyma fibers, contained a significantly higher proportion of mannosyl residues. The adaxial side, however, contained a significantly lower amounts of mannosyl and xylosyl residues, but was enriched in galacturonic acid, rhamnose, galactose and arabinose residues, indicative of primary cell wall components. Noteworthy, we observed similar differences in monosaccharide levels between the three plates in strongly and weakly bending scales (**Supplementary Table S3**).

To further characterize the cell wall composition of the lamina, we quantified crystalline cellulose content across all sample types. This analysis revealed significant difference in crystalline cellulose levels between the VBs and adaxial plate (**Figure 6B**; **Supplementary Tables S1, S2**). The high variation of the cellulose content in the abaxial plate is likely the result of variable contribution in the samples of different cell types characteristic for this plate, namely the sclerenchyma fibers, epidermal and parenchymatous cells.

We, subsequently, examined the cell wall composition in cross sections of the strongly bending lamina region (**Figures 6C–I**). First, we checked the distribution of pectins by staining the sections with propidium iodide (PI), which binds specifically to unesterified galacturonic acid residues (Rounds et al., 2011; Voiniciuc et al., 2018). Staining with PI (confocal microscopy images shown in **Figures 6C–E**) indicates the presence of pectins in all the tissues, including thick walls of fiber-tracheids, tracheids, sclereids, and abaxial sclerenchyma fibers. The observed signal may originate from autofluorescence of lignin, which can be detected also for long emission wavelengths. However, the PI signal is consistent with the monosaccharide content analysis in which galacturonic acid residues were detected in all the three plates. Next, we examined autofluorescence of the cell walls (epi-fluorescence microscopy images shown in **Figures 6F–I**). The UV excitation resulted in strong light blue autofluorescence, which can be assigned to lignin (Donaldson and Williams, 2018), in cell walls of VB xylem fiber-tracheids and tracheids (**Figure 6I**) and of adaxial plate sclereids (**Figure 6F**). Noteworthy, there was an adaxial-abaxial gradient of signal intensity within the VBs, with weaker autofluorescence from the abaxial VB cells. The autofluorescence of walls of the abaxial sclerenchyma fibers and abaxial epidermis (**Figures 6G, H**) was also distinct but weaker than in the VBs.

### 3.5 Tissue plates differ in shape in dry state suggesting that the lamina is a prestressed structure

**Figure 7:**
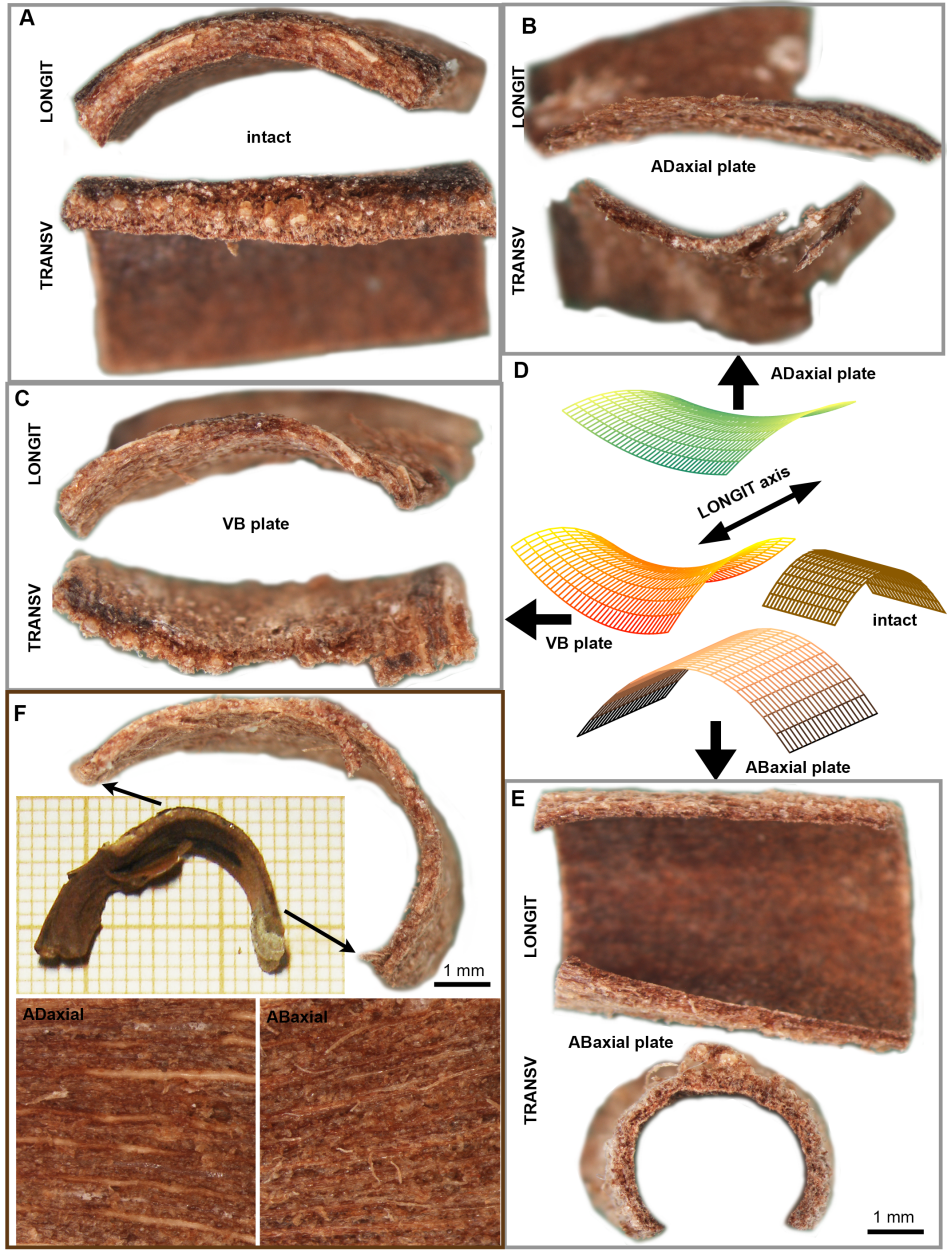
Differential deformation of lamina plates upon drying: **(A)** Dry state profiles of intact lamina square in the direction parallel (LONGIT) and transverse (TRANSV) to the scale axis. **(B–E)** The dry state profiles of three plates **(B, C, E)**, dissected from the lamina square shown in **(A)**, and schematic representation of their geometry **(D)**. **(F)** VB plate dissected from the lamina strip and images of adaxial and abaxial surface of the VB plate. The strip portion from which the plate was dissected is marked by arrows. See the main text for more detailed explanation.

Owing to the differences in deformation and composition between lamina tissues, we focused on the behavior of the three tissue plates, i.e. abaxial, adaxial, and VB plates, upon drying. We dissected the plates from lamina squares (square-shaped lamina fragments cut from the region performing strong hygroscopic movements, **Figures 1C, F**), and compared their shapes in dry state with the shape of intact squares before dissection (**Figure 7A**). The shape of dry VB plate resembles the intact square shape (compare **Figures 7A, C**), in that it is bent (convex) in the longitudinal direction. However, the VB plate is also bent (concave) to a lesser extent in the transverse direction, i.e. it is saddle-shaped (middle scheme in **Figure 7D**). The dry adaxial plate (**Figure 7B**) also has a saddle-like shape; in the longitudinal direction it is slightly convex while in transverse direction it is concave (upper scheme in **Figure 7D**). The shape of dissected abaxial plate is strikingly different from the intact square (compare **Figures 7E, A**, schemes in **Figure 7D**) and the other two plates (compare **Figures 7E, B, C**): the dry abaxial plate is straight in the longitudinal direction and strongly bent (convex) in transverse (bottom scheme in **Figure 7D**). Such the shape differences between the three plates suggest that in an intact dry scale, the tissues are prestressed.

We also dissected the VB plates from lamina strips in order to examine VBs at their entire length, and compared their shape in dry state with the shape of strips from which they were dissected (**Figure 7F**). The dried VB plates are bent in the longitudinal direction. The region of strongest bending is located near the apophysis. This is unlike the intact strip, which is bent mainly near the scale base.

### 3.6 Scale curvature in dry state is related to the lamina thickness and asymmetric position of the VBs in lamina cross section

**Figure 8:**
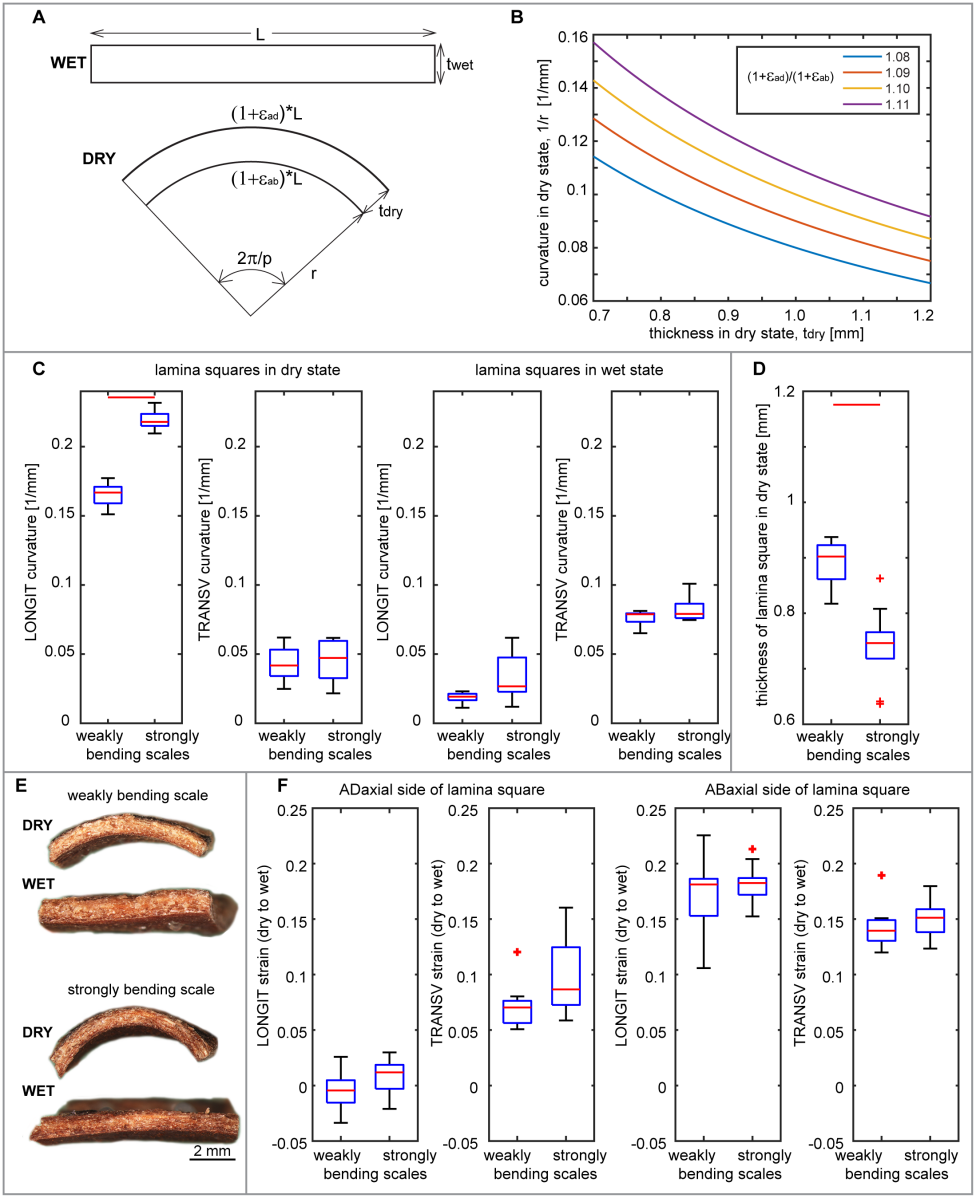
Relationships between lamina thickness, abaxial and adaxial strains, and curvature: **(A)** Schematic representation of longitudinal lamina square profile in wet and dry state, the same as shown in **(E)**, corresponding to the longitudinal side shown in **Figure 1G**. Variables used to compute the theoretical relationships between the lamina square thickness and dry state curvature are: wet square length in the direction parallel to scale axis (L); square thickness in wet (t_wet_) and dry (t_dry_) state; curvature radius of abaxial square surface in dry state (r); longitudinal strains of adaxial (ϵ_ad_) and abaxial (ϵ_ab_) surface during wet to dry transition; portion of full circle used for computation (p). **(B)** Theoretical relationship between dry scale curvature and thickness computed for various ratio of longitudinal strains of abaxial and adaxial surface. **(C)** Curvature of abaxial side of lamina squares in dry and wet state, obtained from weakly and strongly bending scales. Curvature values are means of curvature assessed for points on a curve (second rank polynomial) fitted to the lamina square profile. LONGIT curvature refers to the direction parallel to the scale axis, as shown in **(E)**; TRANSV – to the transverse direction. **(D)** Thickness of weakly and strongly bending scales. **(E)** Longitudinal profiles of exemplary lamina squares obtained from weakly and strongly bending scales, in dry and wet state. **(F)** Longitudinal and transverse strains during transition from dry to wet state assessed for adaxial and abaxial side of squares obtained from weakly and strongly bending scales. See **Figure 2** legend for further explanations. See **Supplementary Table S2** for t-test results.


Next, we focused on questions that have to be addressed at the organ scale. First, we investigated why the differences between adaxial and abaxial surface deformation are similar in scales that are strongly and weakly curved in the dry state (**Figure 8**). We performed simple computation to check how lamina surface strains, size and shape in the dry and wet states are related. We defined strains during the transition from wet to dry state (*ϵ*) as:

$$\epsilon =\frac{L_{dry}-L_{wet}}{L_{wet}}$$

where *L_wet_* and *L_dry_* are lengths in wet and dry state, respectively (**Figure 1G**).

We considered a square-shaped fragment of lamina and assumed that its longitudinal profile is flat in wet state and arc-shaped in dry state [see **Figures 1G**, **8A**; the square profile in **Figure 8A** corresponds to the square side labelled as LONGIT in **Figure 1G**)]. Then, the longitudinal strains of the abaxial and adaxial sides during the transition from wet to dry state can be computed from the following equations:


$$\left(1+\epsilon_{ad}\right)L=\frac{2\pi }{p}\left(r+t_{dry}\right)$$

$$\left(1+\epsilon_{ab}\right)L=\frac{2\pi }{p}r$$

where *ϵ_ad_* and *ϵ_ab_* are longitudinal strains of adaxial and abaxial sides, respectively; *L* is the square length; *t_dry_* – lamina thickness in dry state; *r* – curvature radius in dry state; and *p* – the portion of full circle represented by the arc (**Figure 8A**).

Thus, the ratio of strains on adaxial and abaxial sides is:


$$\frac{1+\epsilon_{ad}}{1+\epsilon_{ab}}=\frac{r+t_{dry}}{r}$$

This simple computation shows that theoretically, the extent of scale lamina bending upon drying depends not only on the strain difference between the two surfaces but also on the lamina thickness, i.e. the distance between the two differentially deforming surfaces (**Figure 8B**). We analyzed these relationships in lamina squares obtained from strongly and weakly bending scales (**Figures 8C, E**). Comparison of thickness of lamina squares showed that indeed the weakly bending scales are significantly thicker than strongly bending ones (**Figure 8D**; **Supplementary Tables S1, S2**) while the deformations of corresponding lamina surfaces are similar (**Figure 8F**; **Supplementary Tables S1, S2**).

**Figure 9:**
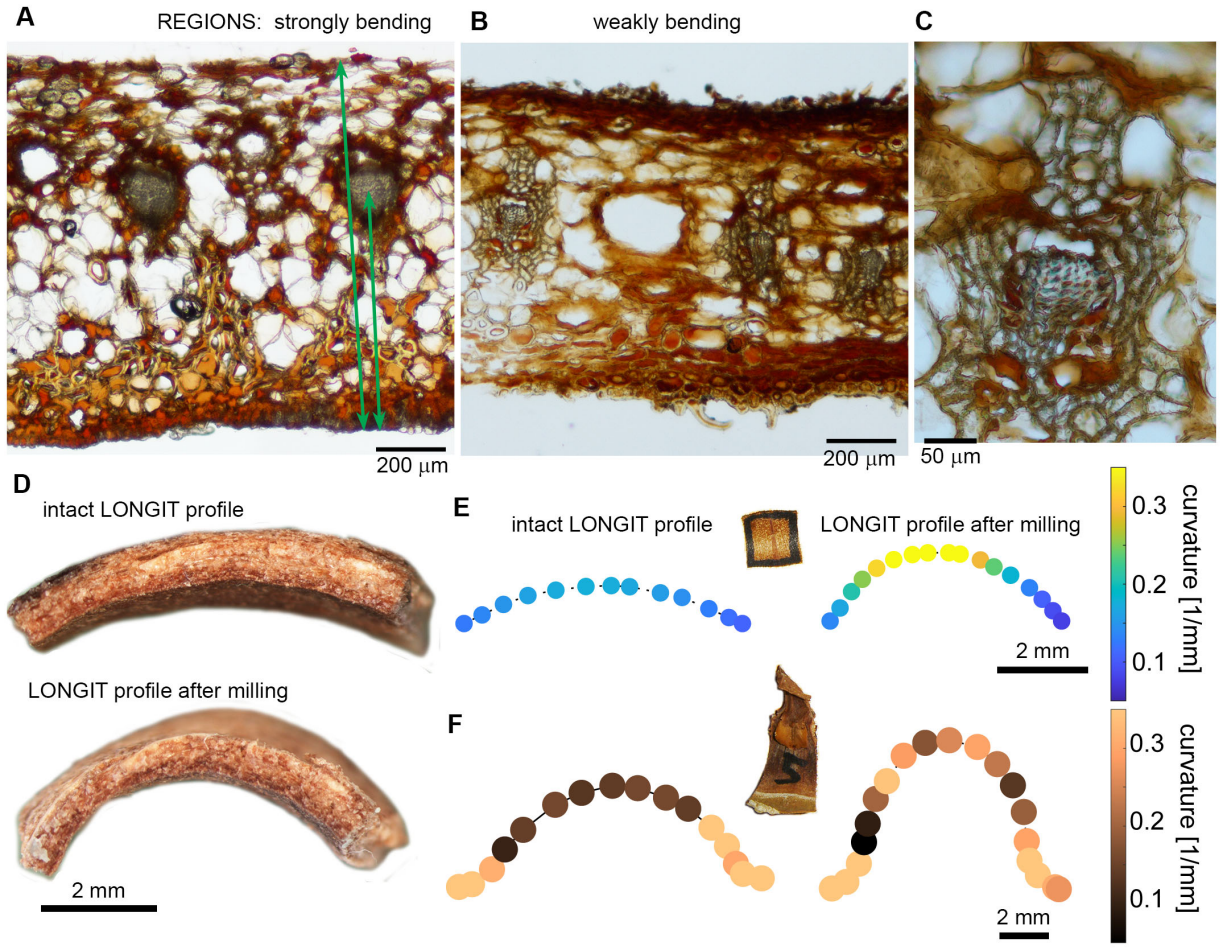
Contribution of adaxial and abaxial plates to the scale lamina thickness and scale curvature in dry state: **(A–C)** Lamina cross-sections obtained from strongly bending proximal/central **(A)** and weakly bending distal **(B)** portions of the same scale, and close-up of VB from the distal portion **(C)**. Distances measured in order to assess the asymmetry of VB position are pointed by arrows. **(D)** Longitudinal profiles of lamina square in dry state, obtained from the weakly bending scale. Note the shape difference between the intact square and the same square after milling of the adaxial plate. **(E, F)** Curvature assessed for points on a curve (second rank polynomial) fitted to longitudinal profiles of lamina square **(E)** and strip **(F)** in dry state, before and after milling of the adaxial plate. **(E)** is the same square as that shown in **(D)**.

**Figure 10:**
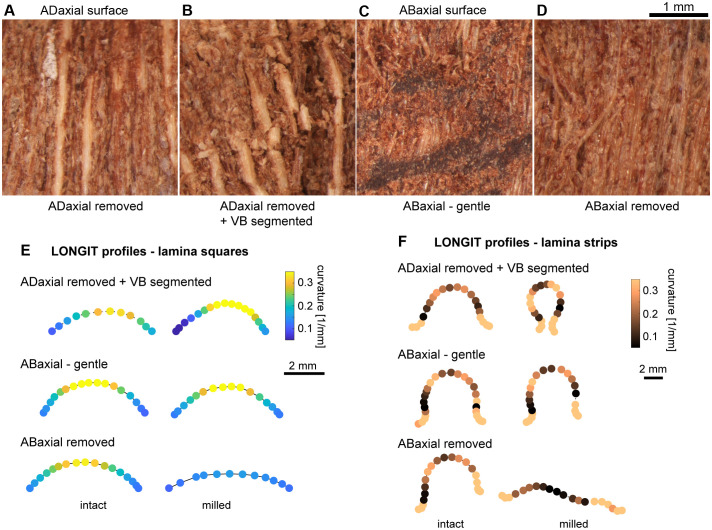
Influence of mechanical damage on the scale bending: **(A–D)** Adaxial or abaxial surface of lamina in dry state after different mechanical damage: **(A)** Deep milling of adaxial plate. Light and thick vertical strips are exposed VBs. **(B)** Deep milling of adaxial plate and segmentation of VBs. Note the broken VBs. **(C)** Gentle milling of abaxial plate. Note the damage of abaxial epidermis. **(D)** Deep milling of abaxial plate. Note the damage of exposed abaxial sclerenchyma fibers. **(E, F)** Curvature assessed for points on a curve (second or third rank polynomial) fitted to longitudinal profiles of lamina squares **(E)** and strips **(F)** in dry state, before and after different mechanical damage: deep milling of adaxial plate and segmentation of VBs (ADaxial removed + VB segmented); gentle milling of abaxial plate (ABaxial – gentle); and deep milling of abaxial plate (ABaxial removed).

Comparisons between the strongly and weakly bending scales revealed a notable difference in the asymmetry of VB plate’s position within the scale cross section (**Figure 9A**). In weakly bending laminas, the location of the VB plate is significantly less asymmetric than in strongly bending ones. In the strongly bending scales, the VBs are closer to the adaxial surface, i.e. the adaxial plate is thinner than in weakly bending scales (**Supplementary Tables S1, S2**). To validate the significance of the location asymmetry of the VB plate, we removed layers of the adaxial tissue by milling (**Figures 9D–F**; see also the adaxial surface after milling in **Figure 10A**). Such thinning of adaxial plate resulted in higher asymmetry of the position of the VB, and indeed led to stronger bending of initially weakly bending scales (compare the two profiles in **Figure 9D** and curvature plots in **Figure 9E**). The milling had similar effect on both lamina squares (**Figure 9E**) and strips (**Figure 9F**). The role of the location of the VB plate in lamina bending is further supported by comparison between the strongly bending lamina portion (**Figure 9A**) and almost straight distal portion of lamina strips (**Figure 9B**). In the distal portion, the VBs are located almost symmetrically, i.e. the thickness of adaxial plate is similar to that of abaxial plate. This may explain why in the case of isolated VB plates, the strongest bending takes place in the distal part, unlike in the case of the intact strips. It is likely that in the intact strip, the distal portion of the VB plate is “deactivated” by relatively thick adaxial plate. Another factor that may be involved in this phenomenon is the specific VB structure (compare **Figures 9C**, **4E, F**), they comprise fewer thick-walled fibers and are surrounded by many more sclereids than VBs in the strongly bending region.

### 3.7 The extent of scale bending in the course of hygroscopic movement depends on relative contribution of the three plates

Knowing that the weakly bending scales can be “activated” by thinning of the adaxial plate, we next attempted to “deactivate” the lamina bending. We checked how various mechanical damage of lamina surface, performed by milling or removal of tissue layers using scalpel (**Figures 10A–D**), affects the lamina curvature in the longitudinal direction in dry state. The experiments were performed on lamina squares (**Figure 10E**) and strips (**Figure 10F**). Because the bending in the longitudinal direction of the VB plate upon drying resembles that of intact lamina, we started from milling the adaxial tissue and breaking the VBs into short segments (**Figure 10B**). Surprisingly this treatment resulted in stronger bending of strips or squares upon drying rather than the “deactivation” (see curvature plot for “ADaxial removed + VB segmented” in **Figures 10E, F**). This pointed to the role of the abaxial plate and continuous VBs in lamina bending. Thus, we damaged the abaxial plate while leaving the adaxial and VB plates intact. First, we performed gentle milling of the abaxial plate surface. Although doing this we damaged the abaxial epidermis (**Figure 10C**) the effect of this treatment was weak and variable (“ABaxial gentle” in **Figures 10E, F**), the curvature of longitudinal profile increased slightly in some dry lamina samples and slightly decreased in others. Then, we removed the abaxial tissue including the entire abaxial epidermis and the majority of abaxial sclerenchyma fibers (**Figure 10D**). This resulted in at least partial deactivation of the lamina bending (“ABaxial removed” in **Figures 10E, F**). Such effect can be explained by the fact that while removing the abaxial tissue we changed the relative position of the VB plate. Overall, the performed treatments reveal the significance of relative contribution of the three plates and also the resilience of the scale as an actuator.

## 4 Discussion

The ovuliferous scales of *Abies concolor* display significant hygroscopic movements. Unlike the pine cone scales, where movement is primarily restricted to the base of the seed-scale complex, *A. concolor* scales undergo deformation across the entire ovuliferous scale lamina. This lamina deformation likely facilitates the detachment of winged seeds from the ovuliferous scale, playing a crucial role in seed dispersal (Aniszewska et al., 2017; Song et al., 2017). In contrast, bending at the base of the seed-scale complex primarily aids in the detachment of the scale from the fir cone axis (Kaniewski and Kucewicz, 1978; Losada et al., 2019).

### 4.1 Both chemical composition of cell walls and tissue structure contribute to the hygroscopic movements of ovuliferous scale of *A. concolor*

The hygroscopic movements are driven by reversible deformation of cell walls in response to changes in water content (Elbaum and Abraham, 2014). Chemical composition and structure of cell walls influence different parameters of this deformation. Swelling ability of cell wall matrix implies the extent of the volumetric expansion (upon wetting) or shrinking (upon drying) of the wall. The main candidate for the swelling component of secondary cell walls is hemicellulose. In pine cone scale, pectins and hemicellulose were detected in cell walls of active and resistance scale layers but only overall hemicellulose content was analyzed (Dawson et al., 1997). Thus, to understand the contribution of cell wall polysaccharides to hydration-driven scale movement it is important to evaluate the properties of the main hemicelluloses present in the sample and to consider their interaction with other cell wall components in wet and dry states. Our HPLC analysis indicates that the abaxial plate, which is the active tissue of the scale lamina, contains large proportion of mannosyl residues. This is likely linked to high content of mannan hemicellulose in this lamina plate. Mannan has strong water-absorbing capacity (Singh et al., 2018) and it is known to be able to reversibly bind and dissociate from the cellulose upon softwood drying and rehydration (Cresswell et al., 2021; Paajanen et al., 2022). On the other hand, xylan, which likely predominates in the VB plate because of high content of xylosyl residues seen in HPLC, has a lower capacity for rehydration than mannan and its association with the cellulose appears to be independent of drying-rehydration cycles (Cresswell et al., 2021). Therefore, our findings suggest that the abaxial side of lamina is rich in polysaccharide moiety whose mobility and interactions with other cell wall components are highly water-dependent. The binding of water at the cell wall matrix level might contribute to the observed scale movement. Importantly the mobility changes of mannan are reversible upon drying and rehydration (Cresswell et al., 2021), which further supports its contribution to the observed phenomenon.

Another key parameter of the cell wall deformation during hygroscopic movements is anisotropy, i.e. directions and values of extremal deformation (maximal and minimal in the case of in-plane deformation of the wall). Cell wall reinforcement by cellulose fibrils affects the deformation anisotropy at the cellular scale (Fratzl et al., 2008; Burgert and Fratzl, 2009). For example, elongated cells with cellulose fibrils nearly perpendicular to the cell axis will tend to extend or shrink mainly along the axis (Borowska-Wykręt et al., 2017). At the tissue scale, the deformation anisotropy depends both on the wall reinforcement of individual cells and on the cell arrangement, i.e. the extent of alignment of elongated cells and the relative cell orientation in adjacent cell layers. For example, resistance tissue of the sesame *Sesamum indicum* capsule comprises two sublayers of mutually orthogonal sclerenchyma fibers, the walls of which are reinforced along the cell axis (Shtein et al., 2016). Such combined sublayers resist very large deformation of the active tissue of the capsule that comprises large thinner-walled parenchyma cells.

The ovuliferous scales of *A. concolor* exhibit relatively simple reinforcement pattern. In mechanical terms the main components of dead tissues are those with the thickest cell walls. In *A. concolor* scale, these thick-walled cells are sclereids of adaxial plate, xylem fiber-tracheids and tracheids of VBs, and sclerenchyma fibers and epidermis of the abaxial plate. Adaxial sclereids are isodiametric cells scattered in parenchyma, which implies isotropic reinforcement. The remaining reinforcing elements, whose walls are relatively rich in crystalline cellulose, are aligned in the direction along the scale axis, suggesting the reinforcement anisotropy. The aligned xylem fiber-tracheids and tracheids are clustered in compact VBs. The abaxial sclerenchyma fibers are also aligned but form a much less compact tissue. On the abaxial surface there is the compact layer of epidermal cells that are also elongated, though to a lesser extent. We were not able to assess directly the wall reinforcement by cellulose fibrils in these different cell types but an indirect information can be drawn from the orientation of slit-like or oval pit apertures (Cockrell, 1974; Lichtenegger et al., 2003). Within the VBs, there is a gradient of cellulose fibril orientation from longitudinal on the adaxial side to transverse on abaxial. A few cells at the abaxial VB boundary have tertiary wall thickenings of helical shape, nearly perpendicular to the tracheid axis. In pine cone scales, similar cells were postulated to generate the driving force of VB movements (Zhang et al., 2022). Such gradient in direction of wall reinforcement together with the gradient of wall density, which decreases in the abaxial direction, explain bending of isolated VBs upon drying. In the abaxial plate, walls of sclerenchyma fibers and large elongated parenchyma cells have transversely oriented fibrils. Such fibril orientation and the high mannan content in the walls explain the strong extension or shrinking of isolated fibers caused by changes in water content. On the other hand, the weakly anisotropic but extensive deformation of isolated abaxial epidermis, coincides with high mannan content and rounded pit apertures that suggest less anisotropic reinforcement of their walls. However, what drives the strongly anisotropic deformation of isolated adaxial tissues remains unclear. The isotropic reinforcement of the adaxial tissue by scattered sclereids may only explain why in the lamina cross-sections, the adaxial plate changes in thickness more than the abaxial plate.

### 4.2 Ovuliferous scales of *A. concolor* undergo distinct hygroscopic movements despite shared structural features with pine cone scales

The majority of studies of the hygroscopic movements of seed cone scales in gymnosperms were performed using pine seed cone as the model. Complementing these studies with fir species characterized by large ovuliferous scales, like *A. concolor*, is advantageous because the entire lamina of the ovuliferous scale is an actuator performing large surface deformation in area and profound shape changes. Our results point to specific structural features of fir scales that can explain these differences between pine and fir scale movements. As already mentioned the morphology of *A. concolor* seed-scale complex differs from pines in the limited fusion of bract and ovuliferous scale in contrast to almost complete fusion in pine. One of the outcomes may be that pine cone scales are thicker and narrower than the fir scales. Such thick pine scales are much less bent in dry state but if ovuliferous scales of fir are thick they are also only weakly bending.

There are important analogies in the structure of pine and fir cone scales. In pine, the actuator comprises abaxial sclereids forming a “skin” and adaxial fibers clustered in compact vascular bundles (Dawson et al., 1997; Zhang et al., 2022). The deformation of abaxial surface of pine cone scale caused by change in hydration is larger than that of the adaxial surface (Eger et al., 2022). Thus, the abaxial skin made of sclereids is regarded as an active tissue. The sclereids are strongly elongated while their walls have nearly transverse cellulose fibrils (Eger et al., 2022) similar to sclerenchyma of the abaxial plate in fir cone scale. Because in fir these sclerenchyma cells fulfil the fiber criteria (Evert, 2006) we use the name ‘abaxial sclerenchyma fibers’. However, the sclereid walls in pine scale are much thicker than walls of abaxial sclerenchyma fibers in fir and the pine sclereids form a compact plate directly connected to the abaxial epidermis (Quan et al., 2021). In the fir cone scale, the abaxial sclerenchyma fibers are scattered between large parenchyma cells and do not form a compact layer adjacent to the abaxial epidermis. These structural traits may be responsible for the strong surface expansion of the abaxial tissue of fir scale, in both longitudinal and transverse directions.

In classic concept, the adaxial VB fibers, with cellulose fibrils oriented nearly parallel to the fiber axis, are the passive (resistance) tissue (Dawson et al., 1997). Later on, closer examination revealed that the structure of pine scale VBs shows adaxial-abaxial gradients of wall density and cellulose fibrils orientation (Quan et al., 2021), as shown for fir VBs in the present investigation. These structural features explain hygroscopic movements of isolated VBs, which occur in both pine and fir. Moreover, in both taxa, the isolated VBs bend stronger in distal than in proximal region likely because of structural gradients along the bundles (Quan et al., 2021; Zhang et al., 2022). Yet another feature shared by pine and fir is the presence of VB fibers (tracheids) with helical tertiary thickenings (Quan et al., 2021; Zhang et al., 2022). Such tracheids (referred to as “spring microtubes” by Zhang et al., 2022) are on the abaxial side of the VBs, adjacent to typical xylem fiber-tracheids (“square microtubes”). Zhang et al. (2022) reported such VB structure for numerous pine (*Pinus*) species and for spruce (*Picea asperata*). They postulated that different behavior of contacting spring and square microtubes upon humidity changes drives the VB bending. This phenomenon, together with gradients of wall density and cellulose fibril orientation, likely contributes to VB bending also in the fir cone scales.

The overall structure of pine cone scale is more complex than a simple bilayer. Firstly, tissue structure is not uniform along the scale axis (Eger et al., 2022), again similar to fir cone scale. For example, in pine, the layer of thick-walled sclereids gradually thins in the distal direction. In both fir and pine, the VBs become thinner towards the scale margin (Eger et al., 2022). Secondly, there is a graded interface between the VBs and abaxial sclereids in pine, made of relatively thin-walled cells with large lumen. It plays a role of a “cushion” minimizing stresses generated by different tissue deformation during humidity changes (Quan et al., 2021). In fir scales, the role of such “cushion” could be played by large parenchyma cells of abaxial plate, embedding groups of sclerenchyma fibers, among which some contact the VBs.

Specific feature of fir scales revealed by our investigations, is the role of the adaxial tissue, i.e. the tissue covering the VB plate on the adaxial side, which we regard as one of three building blocks (plates) of the fir ovuliferous scale. The smaller thickness of the adaxial plate than that of the abaxial plate implies the asymmetric position of VB plate in the fir scale cross-section. Noteworthy, also in pine scale such asymmetric VB position was documented (Quan et al., 2021). In pine, the tissue on the adaxial side of the VBs and between the VBs is the relatively soft “brown tissue” the role of which is currently investigated (Correa et al., 2020; Ulrich et al., 2024).

Other differences between pine and fir cone scales are related to the behavior of the dissected abaxial plate (referred to as a “skin” in pine). In fir, the dissected abaxial plate is strongly bending upon drying and attains the shape entirely different from the VB and adaxial plates, as well as from the intact scale. This is unlike pine, where the dissected “skin” performs hygroscopic movements similar to dissected VB skeleton and intact scale, although the extent of the skin movements is much lower (Quan et al., 2021; Zhang et al., 2022). This difference between fir and pine scales may be related to the structure of the abaxial tissue. It is much more uniform in pine (a plate of compact sclereids closely attached to the epidermis) than in fir, where it is built of fibers scattered in loose parenchyma and covered with compact epidermis.

### 4.3 Hygroscopic movements of ovuliferous scale of *A. concolor* are an emerging property of the fusion of lamina plates

We distinguished three plate-shaped building blocks in the structure of *A. concolor* ovuliferous scale. Boundaries between these plates are not distinct but dissected plates undergo different deformation upon drying: abaxial plate attains a gutter-like shape (**Figure 7E**) while VB (**Figure 7C**) and adaxial (**Figure 7B**) plates are both saddle-shaped but adaxial plate is less curved than the VB plate. Noteworthy, the shapes of all the plates are different from the intact scale lamina shape, unlike the skin and VB skeleton dissected from the pine cone scale (Quan et al., 2021; Zhang et al., 2022; Ulrich et al., 2024). In fir, the adaxial and VB plates are convex in the longitudinal direction similar to the intact lamina but unlike the lamina they are also concave in the transverse direction. The shape of the abaxial plate is completely different: it is straight in the longitudinal direction and strongly convex in the transverse direction, likely because of transverse shrinking of the abaxial epidermis that is not accompanied by transverse shrinking of deeper located fibers and parenchyma. In the intact lamina, *in situ*, during drying such bending of the abaxial plate counteracts the opposite bending of VB and adaxial plates, and in consequence the intact scale is nearly flat in the transverse direction. Thus, an interplay between the differently behaving plates leads to a novel lamina shape that is different than its parts.

Another emerging property of fusion of the three plates is prestressed construction of lamina. The lamina prestresses have different origin than tissue stresses (tissue tensions) in organs built of living cells that are an indirect consequence of turgor (Hejnowicz, 2011). They also differ from so-called growth stresses generated in secondary xylem of trees by shortening of xylem cells during maturation (Romberger et al., 1993). In the case of ovuliferous fir scales, the origin of prestresses is in the different deformation of the lamina plates that accompanies dehydration and is driven exclusively by different structure and composition of dead cell walls. The important consequence of the lamina prestress are likely strong shearing forces at the plate interfaces, especially at the interface of VB and abaxial plates, which in pine are buffered by the already mentioned cushion of large thin-walled cells.

### 4.4 Quantitative variation of lamina Bauplan leads to variation in scale bending and reveals the actuator resilience

An intriguing result obtained during our investigation was the similarity of tissue structure, cell wall composition, and surface strains between ovuliferous scales of *A. concolor* that have quite different shapes in the dry state. However, comparison of lamina thickness and position of VB plate in the lamina cross-section revealed that the extent of lamina bending is related to quantitative variation in these two parameters: the more asymmetric the VB plate position and the thinner the adaxial plate and the entire scale, the stronger is the lamina bending. This rule was further confirmed by the effect of simple manipulation of the adaxial plate thickness. Mechanical thinning of the adaxial plate led to increase in the extent of lamina bending. We postulate that such simple “manipulation” of the lamina Bauplan controls the extent of hygroscopic movements of scales. Such quantitative variation of movement of individual scales could be expected within the cones, and may influence the process of scale shedding.

The mechanical thinning experiments reveal also another important trait of ovuliferous fir scales, i.e. they represent uniquely resilient actuators. The mechanically resilient actuators maintain their capability to perform movements when they are damaged or after a failure event (Perrier et al., 2023; Ulrich et al., 2024). The resilience was studied for pine cone scales by examination of effects of multiple movement cycles, during which failures like delamination or cracks occurred (Ulrich et al., 2024). Despite the failures, the pine cone scales maintained over 90% of movement capability. Another method for resilience assessment is an intentional mechanical damage of the actuator surface. Such experiments where cell layers were removed from the actuator surface, revealed an unusual resilience of the hinge-like actuator of scarious inflorescence bracts of *Helichrysum bracteatum*. This resilience was attributed to the graded construction of the hinge (Borowska-Wykręt et al., 2017). Using a similar method of resilience assessment in the present investigation, we show that mechanical failure of various types affects the hygroscopic movements of ovuliferous scale of *A. concolor* in a quantitative way and often leads to increase in the extent of movement. In particular, damaging of either abaxial or adaxial surface is not able to stop the movements. The movements always increase their extent if adaxial surface is damaged. Even the segmentation of the VBs led to increase in the lamina bending rather than disable it. Apparently, the VB segments are able to participate in movement generation while at the same time the plate with segmented VBs can bend more than the plate with intact ones. The only mechanical damage that nearly disabled the hygroscopic movements was the heavy damage of the abaxial plate (the removal of the entire epidermis and the majority of sclerenchyma fibers). This effect, however, changed the position of VBs within the scale thickness by strongly increasing the contribution of the adaxial plate. Thus, the lamina of ovuliferous scale of *A. concolor* has not only the advantage of being the actuator without a localized hinge, capable of hygroscopic movements over its surface (Speck et al., 2022), it is also resistant to mechanical damage although the damage leads to quantitative changes in the movement. The described construction of the fir cone scale lamina may be an inspiration to design biomimetic actuators similar to what was already proposed for pine cone scales (Correa et al., 2020; Ren et al., 2021). Importantly, the fir scale design offers advantages, thanks to its unique feature of strong expansion and geometry changes of the entire surface (Poppinga et al., 2018; Speck et al., 2022).

## 5 Conclusions

We show that the lamina movements are driven by interplay between three plate-shaped building blocks of the lamina. The plates differ in tissue structure and chemical composition of cell walls. The asymmetry of position of the VB plate within the lamina thickness, which depends on relative contributions of the adaxial and abaxial plates, is crucial for the extent of lamina bending. Therefore, different contribution of the adaxial plate to the scale thickness explains why the scales differ profoundly in the extent of bending, despite their similarities in tissue structure, chemical composition, and surface strains. We postulate that this enables the fir to tune the hygroscopic movements of scales by simple quantitative modifications of the lamina Bauplan. Finally, the lamina of ovuliferous scale of *A. concolor* has two advantages: it is the actuator without a localized hinge and it is resistant to mechanical damage. Fir scale lamina construction may thus be an inspiration to construct biomimetic actuators.

## Data Availability

The original contributions presented in the study are included in the article/**Supplementary Material**. Further inquiries can be directed to the corresponding authors.

## References

[B1] Aniszewska M. Gendek A. Śliwińska J. (2017). Variability of silver fir (Abies alba Mill.) cones – variability structure of scale surface area. For. Res. Pap. 78, 5–13. doi: 10.1515/frp-2017-0001

[B2] Armon S. Efrati E. Kupferman R. Sharon E. (2011). Geometry and mechanics in the opening of chiral seed pods. Science 333, 1726–1730. doi: 10.1126/science.1203874 21940888

[B3] Barbier de Reuille P. Routier-Kierzkowska A. L. Kierzkowski D. Bassel G. W. Schüpbach T. Tauriello G.. (2015). MorphoGraphX: A platform for quantifying morphogenesis in 4D. eLife 4, e05864. doi: 10.7554/elife.05864 25946108 PMC4421794

[B4] Borowska-Wykręt D. Rypień A. Dulski M. Grelowski M. Wrzalik R. Kwiatkowska D. (2017). Gradient of structural traits drives hygroscopic movements of scarious bracts surrounding Helichrysum bracteatum capitulum. Ann. Bot. 119, 1365–1383. doi: 10.1093/aob/mcx015 28334385 PMC5604587

[B5] Burgert I. Fratzl P. (2009). Plants control the properties and actuation of their organs through the orientation of cellulose fibrils in their cell walls. Integr. Comp. Biol. 49, 69–79. doi: 10.1093/icb/icp026 21669847

[B6] Cockrell R. A. (1974). A comparison of latewood pits, fibril orientation, and shrinkage of normal and compression wood of Giant Sequoia. Wood Sci. Technol. 8, 197–206. doi: 10.1007/bf00352023

[B7] Correa D. Poppinga S. Mylo M. D. Westermeier A. S. Bruchmann B. Menges A.. (2020). 4D pine scale: biomimetic 4D printed autonomous scale and flap structures capable of multi-phase movement. Phil. Trans. R. Soc A. 378, 20190445. doi: 10.1098/rsta.2019.0445 32008450 PMC7015286

[B8] Cresswell R. Dupree R. Brown S. P. Pereira C. S. Skaf M. S. Sorieul M.. (2021). Importance of water in maintaining softwood secondary cell wall nanostructure. Biomacromolecules 22, 4669–4680. doi: 10.1021/acs.biomac.1c00937 34669375 PMC8579401

[B9] Dawson C. Vincent J. F. Rocca A.-M. (1997). How pine cones open. Nature 390, 668–668. doi: 10.1038/37745

[B10] Donaldson L. Williams N. (2018). Imaging and spectroscopy of natural fluorophores in pine needles. Plants 7, 10. doi: 10.3390/plants7010010 29393922 PMC5874599

[B11] Eger C. J. Horstmann M. Poppinga S. Sachse R. Thierer R. Nestle N.. (2022). The structural and mechanical basis for passive-hydraulic pine cone actuation. Adv. Sci. 9, 2200458. doi: 10.1002/advs.202200458 PMC9284161 35567337

[B12] Elbaum R. Abraham Y. (2014). Insights into the microstructures of hygroscopic movement in plant seed dispersal. Plant Sci. J. 223, 124–133. doi: 10.1016/j.plantsci.2014.03.014 24767122

[B13] Elbaum R. Gorb S. Fratzl P. (2008). Structures in the cell wall that enable hygroscopic movement of wheat awns. J. Struct. Biol. 164, 101–107. doi: 10.1016/j.jsb.2008.06.008 18625323

[B14] Evert R. F. (2006). Esau’s plant anatomy: meristems, cells, and tissues of the plant body: their structure, function, and development (Hoboken, New Jersey: John Wiley & Sons, Inc. Publication). doi: 10.1002/0470047380

[B15] Fang L. Ishikawa T. Rennie E. A. Murawska G. M. Lao J. Yan J.. (2016). Loss of inositol phosphorylceramide sphingolipid mannosylation induces plant immune responses and reduces cellulose content in arabidopsis. Plant Cell. 28, 2991–3004. doi: 10.1105/tpc.16.00186 27895225 PMC5240734

[B16] Forterre Y. Dumais J. (2011). Generating helices in nature. Science 333, 1715–1716. doi: 10.1126/science.1210734 21940886

[B17] Fratzl P. Elbaum R. Burgert I. (2008). Cellulose fibrils direct plant organ movements. Faraday Discuss. 139, 275–282. doi: 10.1039/b716663j 19049001

[B18] Goodall C. R. Green P. B. (1986). Quantitative analysis of surface growth. Bot. Gaz. 147, 1–15. doi: 10.1086/337562

[B19] Goubet F. Barton C. J. Mortimer J. C. Yu X. Zhang Z. Miles G. P.. (2009). Cell wall glucomannan in Arabidopsis is synthesised by CSLA glycosyltransferases, and influences the progression of embryogenesis. Plant J. 60, 527–538. doi: 10.1111/j.1365-313x.2009.03977.x 19619156

[B20] Hejnowicz Z. (2011). “Plants as mechano-osmotic transducers,” in Mechanical integration of plant cells and plants. Ed. Wojtaszek P. (Springer-Verlag, Heidelberg).

[B21] Kaniewski K. Kucewicz O. (1978). Anatomical development of the *Abies alba* Mill. cone and shedding of its scales during ripening. Zeszyty Naukowe SGGW w Warszawie Leśnictwo. 26, 141–158.

[B22] Kolotelo D. (1997). Anatomy & Morphology of conifer tree seed (British Columbia: Ministry of Forest, Nursery and Seed Operations Branch).

[B23] Kumar M. Turner S. (2015). Protocol: A medium-throughput method for determination of cellulose content from single stem pieces of Arabidopsis thaliana. Plant Methods 11, 46. doi: 10.1186/s13007-015-0090-6 26464578 PMC4603690

[B24] Lichtenegger H. C. Müller M. Wimmer R. Fratzl P. (2003). Microfibril angles inside and outside Crossfields of Norway Spruce Tracheids. Holzforschung 57, 13–20. doi: 10.1515/hf.2003.003

[B25] Liszka A. Wightman R. Latowski D. Bourdon M. Krogh K. B. Pietrzykowski M.. (2023). Structural differences of cell walls in Earlywood and latewood of Pinus sylvestris and their contribution to biomass recalcitrance. Front. Plant Sci. 14. doi: 10.3389/fpls.2023.1283093 PMC10749964 38148867

[B26] Losada J. M. Blanco-Moure N. Leslie A. B. (2019). Not all “pine cones” flex: Functional trade-offs and the evolution of seed release mechanisms. New Phytol. 222, 396–407. doi: 10.1111/nph.15563 30367490

[B27] Paajanen A. Zitting A. Rautkari L. Ketoja J. A. Penttilä P. A. (2022). Nanoscale mechanism of moisture-induced swelling in wood microfibril bundles. Nano Lett. 22, 5143–5150. doi: 10.1021/acs.nanolett.2c00822 35767745 PMC9284609

[B28] Perrier R. Tadrist L. Linares J.-M. (2023). Damage resilience of manufactured and biological actuators. Bioinspir. Biomim. 18, 016006. doi: 10.1088/1748-3190/ac9fb6 36322997

[B29] Poppinga S. Zollfrank C. Prucker O. Rühe J. Menges A. Cheng T.. (2018). Toward a new generation of smart biomimetic actuators for architecture. Adv. Mater. 30, 1703653. doi: 10.1002/adma.201703653 29064124

[B30] Quan H. Pirosa A. Yang W. Ritchie R. O. Meyers M. A. (2021). Hydration-induced reversible deformation of the pine cone. Acta Biomater. 128, 370–383. doi: 10.1016/j.actbio.2021.04.049 33964479

[B31] Ren L. Li B. Wang K. Zhou X. Song Z. Ren L.. (2021). Plant-morphing strategies and plant-inspired soft actuators fabricated by biomimetic four-dimensional printing: A review. Front. Mater. 8. doi: 10.3389/fmats.2021.651521

[B32] Reyssat E. Mahadevan L. (2009). Hygromorphs: from pine cones to biomimetic bilayers. J. R. Soc Interface. 6, 951–957. doi: 10.1098/rsif.2009.0184 19570796 PMC2838359

[B33] Romberger J. A. Hejnowicz Z. Hill J. F. (1993). Plant structure: function and development (Berlin: Springer-Verlag). doi: 10.1007/978-3-662-01662-6

[B34] Rounds C. M. Lubeck E. Hepler P. K. Winship L. J. (2011). Propidium iodide competes with Ca^2+^ to label pectin in pollen tubes and Arabidopsis root hairs. Plant Physiol. 157, 175–187. doi: 10.1104/pp.111.182196 21768649 PMC3165868

[B35] Shtein I. Elbaum R. Bar-On B. (2016). The hygroscopic opening of sesame fruits is induced by a functionally graded pericarp architecture. Front. Plant Sci. 7. doi: 10.3389/fpls.2016.01501 PMC5056167 27777579

[B36] Singh S. Singh G. Arya S. K. (2018). Mannans: An overview of properties and application in food products. Int. J. Biol. Macromol. 119, 79–95. doi: 10.1016/j.ijbiomac.2018.07.130 30048723

[B37] Song K. Chang S.-S. Lee S. J. (2017). How the pine seeds attach to/detach from the Pine Cone Scale. Front. Life Sci. 10, 38–47. doi: 10.1080/21553769.2017.1287777 29732239 PMC5935463

[B38] Song K. Yeom E. Seo S.-J. Kim K. Kim H. Lim J.-H.. (2015). Journey of water in pine cones. Sci. Rep. 5, 9963. doi: 10.1038/srep09963 25944117 PMC4421802

[B39] Speck T. Poppinga S. Speck O. Tauber F. (2022). Bio-inspired life-like motile materials systems: Changing the boundaries between living and technical systems in the Anthropocene. Anthropocene Rev. 9, 237–256. doi: 10.1177/20530196211039275

[B40] Ulrich K. Genter L. Schäfer S. Masselter T. Speck T. (2024). Investigation of the resilience of cyclically actuated pine cone scales of Pinus jeffreyi. Bioinspir. Biomim. 19, 046009. doi: 10.1088/1748-3190/ad475b 38701824

[B41] Voiniciuc C. Pauly M. Usadel B. (2018). Monitoring polysaccharide dynamics in the plant cell wall. Plant Physiol. 176, 2590–2600. doi: 10.1104/pp.17.01776 29487120 PMC5884611

[B42] Yu L. Shi D. Li J. Kong Y. Yu Y. Chai G.. (2014). CELLULOSE SYNTHASE-LIKE A2, a glucomannan synthase, is involved in maintaining adherent mucilage structure in Arabidopsis seed. Plant Physiol. 164, 1842–1856. doi: 10.1104/pp.114.236596 24569843 PMC3982747

[B43] Zhang F. Jiang L. Yang M. Xu X. Liu X. Liu H.. (2022). Unperceivable motion mimicking hygroscopic geometric reshaping of pine cones. Nat. Mater. 21, 1357–1365. doi: 10.1038/s41563-022-01391-2 36357689

