# Supplementary material for: Hygroscopic movements of cone scale of white fir Abies concolor are tuned by quantitative variation of the scale Bauplan
Source: Front Plant Sci. 2025 May 30;16:1603330. doi: 10.3389/fpls.2025.1603330 (PMC12162492; doi:10.3389/fpls.2025.1603330)
Supplement: Supplementary file 1 [file Presentation1.pdf]

Supplementary Material

1 Supplementary Figures and Tables

1.1 Supplementary Figures

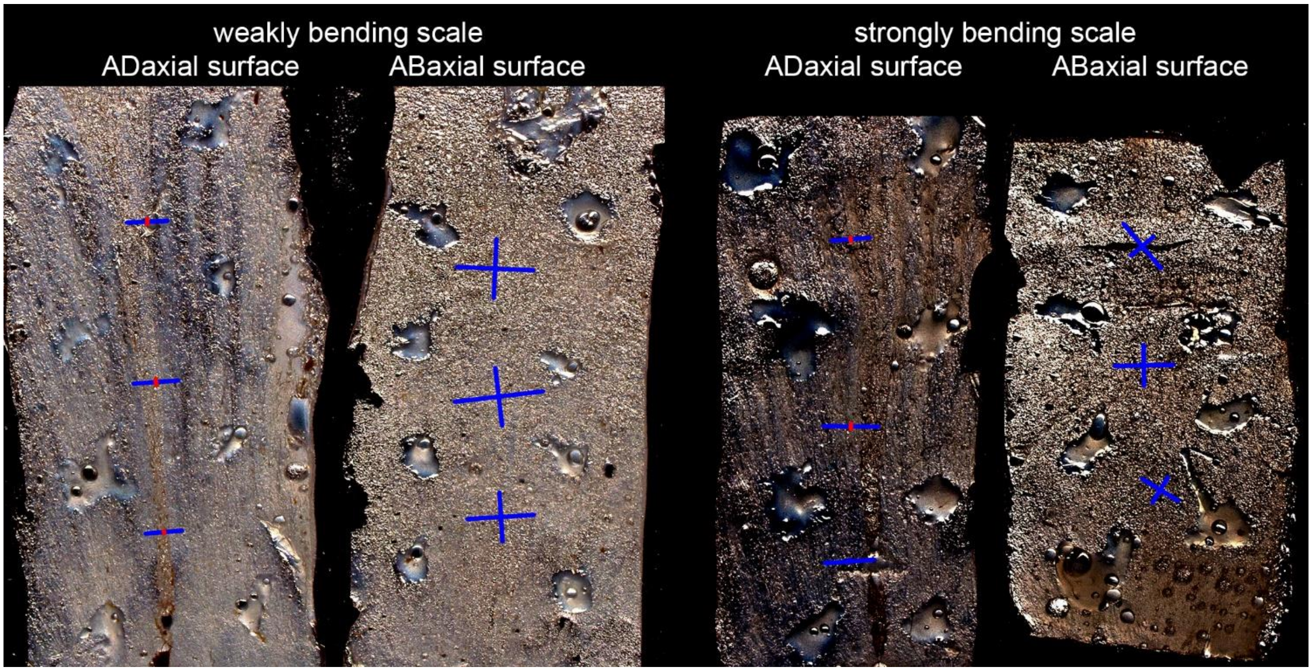

Supplementary Figure 1. Original images of nail polish replicas obtained from both the surfaces of two individual scale strips with overlaid maps of deformation during transition from dry to wet state. Crosses show directions of maximal and minimal deformation, the arm lengths are proportional to strain in the arm direction, blue color represents extension, red – shrinking.

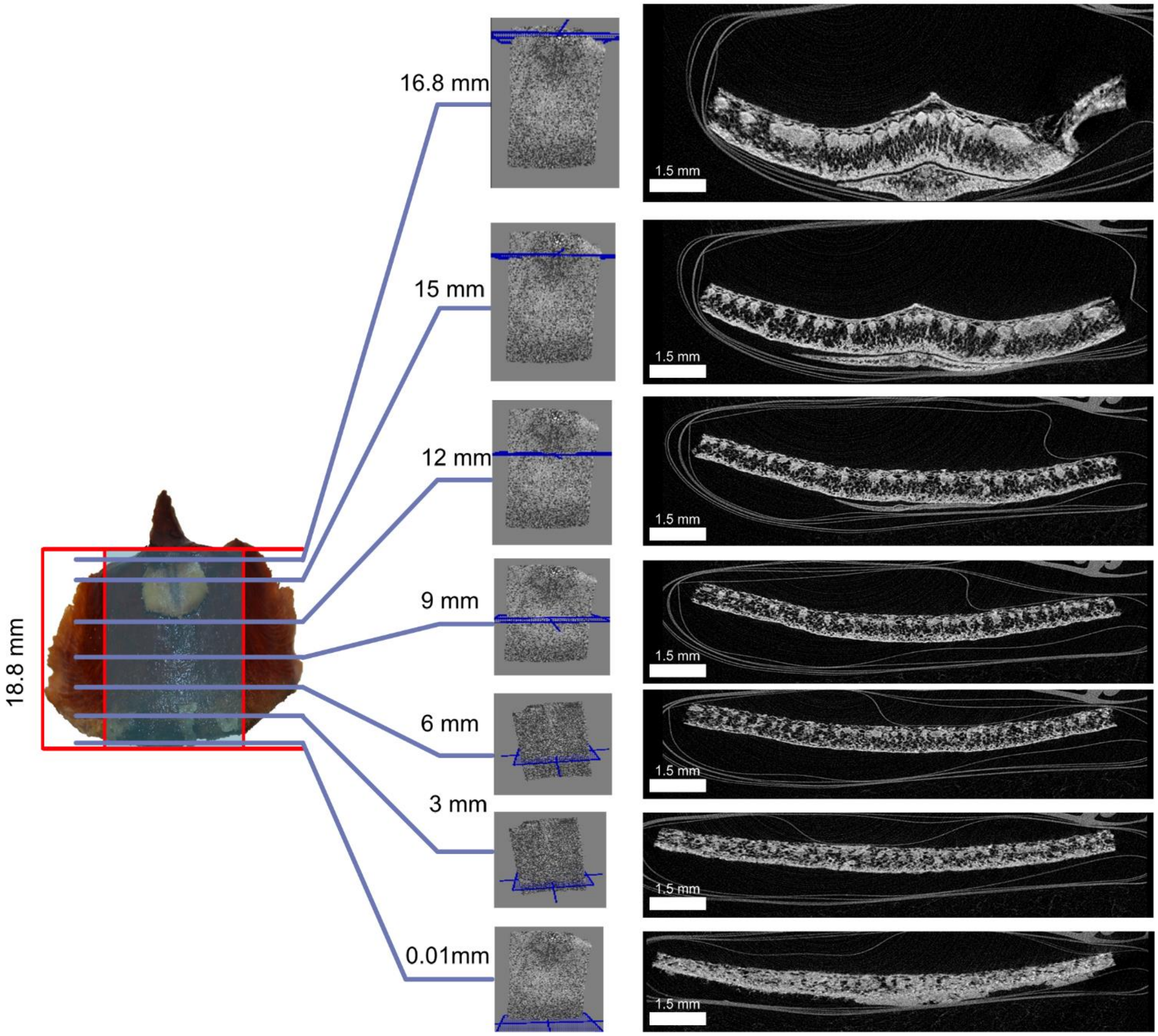

Supplementary Figure 2. Serial cross-sections of ovuliferous scale, transverse to the scale axis, obtained at different distance from the scale distal margin from the μCT 3D reconstruction model of scale strip. In order to avoid drying the sample was wrapped in plastic foil, which is visible in images as thin outlines.

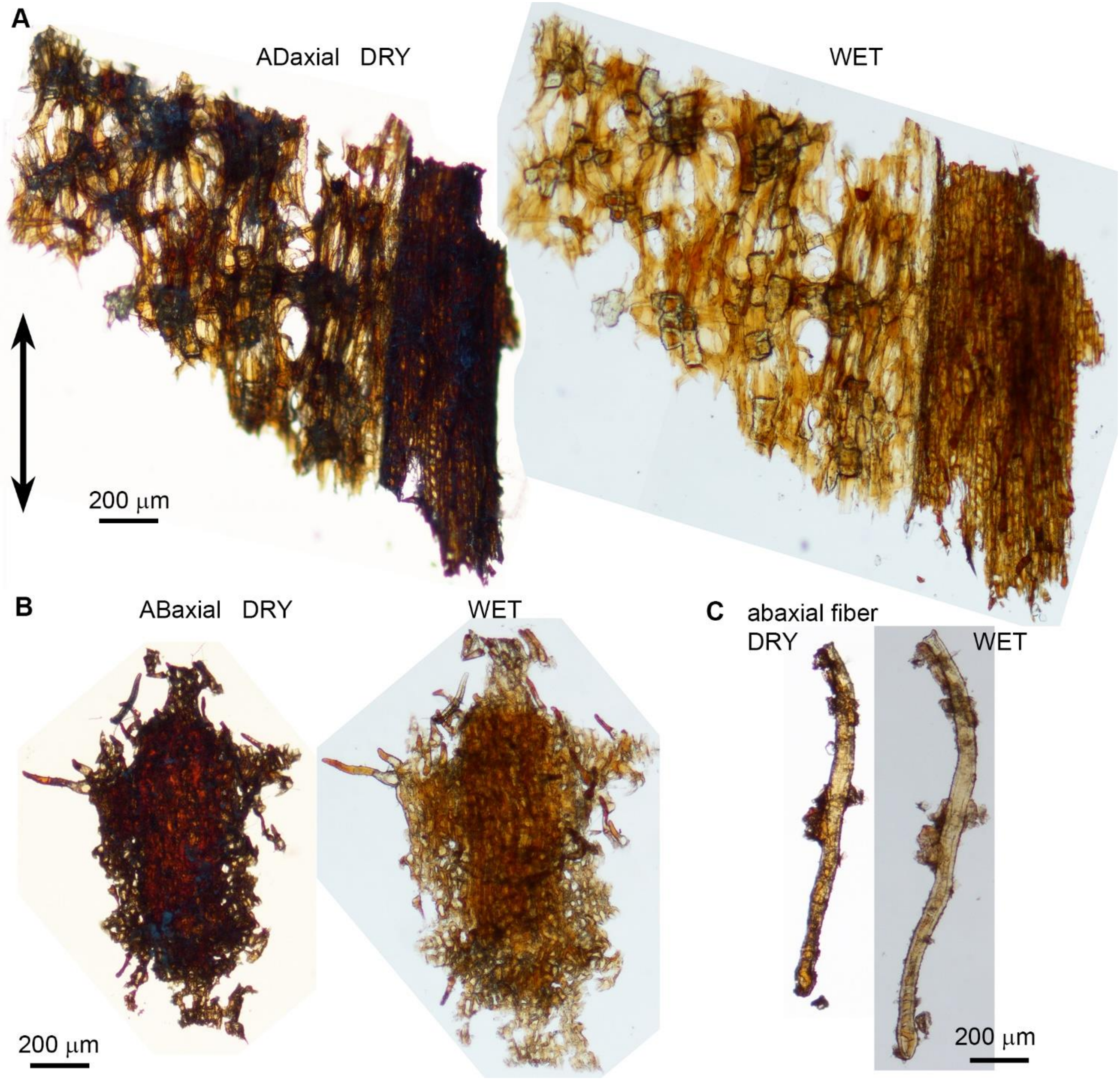

Supplementary Figure 3. Exemplary isolated tissues in dry and wet states that were used to assess strains in longitudinal and transverse direction: **(A)** Paradermal section of adaxial scale surface. **(B)** Paradermal section of abaxial scale surface. **(C)** Abaxial sclerenchyma fiber. All samples are oriented along the long scale axis, pointed by the line segment with arrows.

1.2 Supplementary Tables

Supplementary Table 1. Descriptive statistics of parameters shown in Figures 2, 5, 6, 8, 9. See Figure legends for further explanation. SD – standard deviation; n – number of measurements

	weakly bending scales			strongly bending scales		
	mean	SD	n	mean	SD	n
dry to wet strains of strips assessed from replicas (Figure 2):						
ADaxial LONGIT	-0.02	0.02	10	-0.05	0.03	12
ADaxial TRANSV	0.11	0.04	10	0.12	0.03	12
ABaxial LONGIT	0.12	0.03	10	0.14	0.04	12
ABaxial TRANSV	0.22	0.02	10	0.22	0.24	12
dry to wet strains in thickness of cross-section (Figure 5B):						
scale #1 AB-AD	0.20	0.04	30	0.19	0.04	11
scale #1 AB-VB	0.17	0.04	30	0.19	0.05	11
scale #2 AB-AD	0.24	0.03	24	0.24	0.03	19
scale #2 AB-VB	0.16	0.03	24	0.23	0.03	19
scale #3 AB-AD	0.22	0.05	17	0.21	0.03	22
scale #3 AB-VB	0.19	0.05	17	0.17	0.03	22
dry to wet strains assessed from square profiles (Figure 8F):						
ADaxial LONGIT	-0.01	0.02	5	0.01	0.02	5
ADaxial TRANSV	0.07	0.02	5	0.10	0.03	5
ABaxial LONGIT	0.17	0.03	5	0.18	0.02	5
ABaxial TRANSV	0.14	0.02	5	0.15	0.02	5
curvature of square profile in dry state [mm^{-1}] (Figure 8):						
LONGIT	0.17	0.01	5	0.22	0.01	5
TRANSV	0.04	0.01	5	0.05	0.02	5
curvature of square profile in wet state [mm^{-1}] (Figure 8):						
LONGIT	0.02	0.00	5	0.03	0.02	5
TRANSV	0.08	0.01	5	0.08	0.01	5
thickness of lamina square in dry state [mm] (Figure 8D)	0.89	0.04	5	0.74	0.07	5
relative position of VB in scale cross section [%] (Figure 9)	62.08	1.08	4	67.7	4.25	4

	strongly bending scales		
	mean	SD	n
dry to wet strains of isolated tissues (Figure 5C-E):			
ADaxial LONGIT	0.06	0.02	17
ADaxial TRANSV	0.16	0.06	17
ABaxial LONGIT	0.22	0.03	23
ABaxial TRANSV	0.26	0.04	23
abaxial fibers (LONGIT)	0.20	0.06	14
VB ADaxial side	-0.03	0.02	5
VB ABaxial side	0.09	0.04	5
Figure 6B:			
crystalline cellulose in AB [µg/mg AIR]	142.52	35.23	3
crystalline cellulose in VB [µg/mg AIR]	176.68	10.24	3
crystalline cellulose in AD [µg/mg AIR]	133.50	12.43	3

Supplementary Table 2. Results of *t*-test pair-wise comparisons of parameters shown in Figures 2, 5, 6, 8, 9. See Figure legends for further explanation. *p* values lower than 0.05 are in red.

compared samples	*p* value of *t*-test
weakly vs. strongly bending scales	
dry to wet strains of strips assessed from replicas (Figure 2):	
ADaxial LONGIT	0.03
ADaxial TRANSV	0.62
ABaxial LONGIT	0.32
ABaxial TRANSV	0.86
dry to wet strains assessed from square profiles (Figure 8):	
ADaxial LONGIT	0.08
ADaxial TRANSV	0.06
ABaxial LONGIT	0.42
ABaxial TRANSV	0.43
curvature of square profile in dry state (Figure 8):	
LONGIT	< 0.0001
TRANSV	0.85
curvature of square profile in wet state (Figure 8):	
LONGIT	0.12
TRANSV	0.87
lamina square thickness in dry state (Figure 8)	< 0.0001
relative position of VB in scale cross section (Figure 9)	0.04
proximal vs distal strip region	
dry to wet strains of strips assessed from replicas (Figure 2):	
ADaxial LONGIT	0.03
ADaxial TRANSV	0.03
ABaxial LONGIT	0.06
ABaxial TRANSV	0.01
central vs distal strip region	
dry to wet strains of strips assessed from replicas (Figure 2):	
ADaxial LONGIT	0.02
ADaxial TRANSV	0.04
ABaxial LONGIT	0.09
ABaxial TRANSV	0.02
other comparisons	
dry to wet strains of isolated tissues (Figure 5):	
ADaxial LONGIT vs ABaxial LONGIT	< 0.0001
ADaxial TRANSV vs ABaxial TRANSV	< 0.0001
dry to wet strains in thickness of cross-section - AB-AD vs AB-VB (Figure 5):	
strongly bending scale #1	0.86
strongly bending scale #2	0.49
strongly bending scale #3	< 0.0001
weakly bending scale #1	0.001
weakly bending scale #2	< 0.0001
weakly bending scale #3	0.04
Figure 6:	
Crystalline cellulose ABaxial vs VB plates	0.15
Crystalline cellulose ABaxial vs ADaxial plates	0.58
Crystalline cellulose VB vs ADaxial plates	0.01
